# Frequent and recent retrotransposition of orthologous genes plays a role in the evolution of sperm glycolytic enzymes

**DOI:** 10.1186/1471-2164-11-285

**Published:** 2010-05-06

**Authors:** Soumya A Vemuganti, Fernando Pardo-Manuel de Villena, Deborah A O'Brien

**Affiliations:** 1 Department of Cell and Developmental Biology, University of North Carolina School of Medicine, Chapel Hill, NC 27599, USA; 2 Department of Genetics, Carolina Center for Genome Sciences, University of North Carolina School of Medicine, Chapel Hill, NC 27599, USA; 3 Laboratories for Reproductive Biology, Department of Pediatrics, University of North Carolina School of Medicine, Chapel Hill, NC 27599, USA; 4 Lineberger Comprehensive Cancer Center, University of North Carolina School of Medicine, Chapel Hill, NC 27599, USA

## Abstract

**Background:**

The central metabolic pathway of glycolysis converts glucose to pyruvate, with the net production of 2 ATP and 2 NADH per glucose molecule. Each of the ten reactions in this pathway is typically catalyzed by multiple isozymes encoded by a multigene family. Several isozymes in this pathway are expressed only during spermatogenesis, and gene targeting studies indicate that they are essential for sperm function and male fertility in mouse. At least three of the novel glycolytic isozymes are encoded by retrogenes (*Pgk2*, *Aldoart1*, and *Aldoart2*). Their restricted expression profile suggests that retrotransposition may play a significant role in the evolution of sperm glycolytic enzymes.

**Results:**

We conducted a comprehensive genomic analysis of glycolytic enzymes in the human and mouse genomes and identified several intronless copies for all enzymes in the pathway, except *Pfk*. Within each gene family, a single orthologous gene was typically retrotransposed frequently and independently in both species. Several retroposed sequences maintained open reading frames (ORFs) and/or provided evidence of alternatively spliced exons. We analyzed expression of sequences with ORFs and <99% sequence identity in the coding region and obtained evidence for the expression of an alternative *Gpi1 *transcript in mouse spermatogenic cells.

**Conclusions:**

Our analysis detected frequent, recent, and lineage-specific retrotransposition of orthologous glycolytic enzymes in the human and mouse genomes. Retrotransposition events are associated with LINE/LTR and genomic integration is random. We found evidence for the alternative splicing of parent genes. Many retroposed sequences have maintained ORFs, suggesting a functional role for these genes.

## **Background**

Although glycolysis is highly conserved, this central metabolic pathway is modified extensively during spermatogenesis. There are several glycolytic isozymes with restricted expression in the male germline including spermatogenic glyceraldehyde-3-phosphate dehydrogenase (GAPDHS) [1,2], phosphoglycerate kinase 2 (PGK2) [3], and two aldolase A(ALDOA)-related isozymes (ALDOART1 and ALDOART2) in mouse [4]. Other unique sperm isozymes in this pathway are generated by alternative splicing, including hexokinase 1 variants (HK1_V1 and HK1_V2) [5-7], ALDOA_V2 [4], and a pyruvate kinase muscle form isozyme (PK-S) [8]. There is also evidence that other glycolytic enzymes have unique functional or structural properties in mammalian sperm, including glucose phosphate isomerase (GPI1) [9,10], triose phosphate isomerase (TPI) [11], enolase (ENO) [12-14], and phosphofructokinase (PFK) [15].

Sperm motility is dependent upon the production of high levels of ATP in the flagellum [16-18]. Targeted disruption of genes encoding two spermatogenic cell-specific glycolytic enzymes (*Gapdhs *and *Pgk2*) demonstrates an essential role of these enzymes in sperm motility and male fertility in mice [19,20]. *Ldhc*, which encodes a germ cell-specific LDH isozyme for the conversion of pyruvate to lactate, is also required [21]. A recent study of 1085 patients with male factor infertility found that approximately 81% exhibit defects in sperm motility, with 19% having no other defects in sperm count or morphology [22]. The expression of genes that promote high sperm motility can increase reproductive fitness, while disruptive mutations in genes essential for sperm motility can hinder proper fertilization, leading to infertility. In humans, genes involved in spermatogenesis and sperm motility demonstrate the strongest evidence for positive selection, and proteins involved in reproduction are among the most rapidly evolving genes across multiple species [23,24].

The glycolytic pathway is comprised of ten enzymes, each encoded by a multigene family [25]. Seven of these gene families have two to five intron-containing genes, while the *Gpi1*, *Tpi1*, and *Pgk *families each have only one. Within a family, each gene encodes a different isoform with a unique expression pattern [25]. Many of these gene families arose by multiple rounds of segmental gene duplication in the last 150 million years [25]. Genes encoding spermatogenic cell-specific glycolytic isozymes were generated by either segmental gene duplication (*Gapdhs*) or retrotransposition (*Pgk2, Aldoart1, Aldoart2*) [3,4,26,27]. *Pgk2 *represent an ancient retrotransposition event shared by all eutherian mammals, while *Aldoart1 *and *Aldoart2 *are only found in the rodent lineage and are much more recent [4,28]. In addition, frequent retrotransposition of the *Gapdh *and *Aldoa *genes has been reported in both human and mouse, based on an abundance of pseudogenes [29-32].

Theoretically, retrotransposition can occur in any cell type, but the retrotransposition event is only transmitted to future generations when it takes place in the germline [33-36]. Retrotransposition is facilitated by repetitive elements (including LINE and LTR elements), resulting in the creation of pseudogenes or retrogenes [37]. In the human lineage most LTR elements have been extinct for over 40 million years. However, LINE elements are still active and are, therefore, thought to be responsible for most retroposed mRNA sequences [37]. The proteins encoded by LINE elements provide both endonuclease and reverse transcriptase activities required for retrotransposition. These proteins, ORF1 and ORF2, are expressed in testicular germ cells undergoing meiosis, a period when retrotransposition is thought to occur [34,36,38]. In fact, retrotransposition is responsible for the creation of many retrogenes expressed only during the meiotic and/or haploid phases of spermatogenesis, including but not limited to *Pgk2*, *G6pd2*, and *Pabp2 *[3,26,39,40]. At least 10% of retroposed sequences with open reading frames (ORF) are transcribed during spermatogenesis [38,41-43]. Positive selection of sperm proteins, combined with frequent retrotransposition to create genes encoding sperm-specific proteins, results in the faster evolution of genes involved in sperm function [44].

Based on the existence of *Pgk2 *and *Aldoa-*related retrogenes and their restricted expression during spermatogenesis, we hypothesized that there may be additional retrogenes that encode novel sperm glycolytic enzymes. Therefore, we conducted a comprehensive genomic analysis to identify all human and mouse retroposed sequences that are derived from genes encoding glycolytic enzymes. We analyzed the gene structure of these sequences and determined which copies maintain ORFs, are transcribed, and may encode sperm-specific isoforms of glycolytic enzymes. Unique features of sperm glycolytic isozymes may be important for localization of this pathway in the principal piece of the sperm flagellum or for altered regulation or kinetic properties that may be required to sustain sperm metabolism and motility in this highly polarized cell. Taken together, identification of all sperm-specific glycolytic enzymes will improve our understanding of sperm metabolism at a molecular level and may provide insights regarding the rapid evolution of genes required for reproduction.

## Results

### Frequent retrotransposition of orthologous genes encoding glycolytic enzymes occurred independently in the mouse and human genomes

There are 25 intron-containing genes in the ten gene families that compose the glycolytic pathway (Table 1). We used BLAST to identify sequences with significant sequence similarity to each parent gene (see Methods for details). This analysis identified retroposed sequences in the human and mouse genomes in every family of glycolytic enzymes, except phosphofructokinase (*Pfk)*. Major conclusions from this analysis are:

**Table 1:** Gene families encoding glycolytic enzymes and the parent genes that are retroposed.

	Human			Mouse		
		
Gene Family	Gene Name	Position	**Ret.seq**.	Gene Name	Position	**Ret.seq**.
Hexokinase	*HK1*	10:71	No	***Hk1***	10:62	Yes (1)
	***HK2***	02:75	Yes (1)	*Hk2*	6:83	No
	*HK3*	05:176	No	*Hk3*	13:55	No
	*GCK*	07:44	No	*Gck*	11:6	No
	*HKDC1*	10:71	No	*Hkdc1*	10:62	No

Glucose Phosphate Isomerase	***GPI1***	19:40	Yes (1)	***Gpi1***	7:34	Yes (1)

Phosphofructokinase	*PFKL*	21:45	No	*Pfkl*	10:77	No
	*PFKM*	12:47	No	*Pfkm*	15:98	No
	*PFKP*	10:3	No	*Pfkp*	13:7	No

Aldolase	***ALDOA***	16:30	Yes (2)	***Aldoa***	7:127	Yes (18)
	*ALDOB*	09:103	No	*Aldob*	4:50	No
	*ALDOC*	17:24	No	*Aldoc*	11:78	No

Triosephosphate isomerase	***TPI1***	12:7	Yes (4)	***Tpi1***	6:125	Yes (15)

Glyceraldehyde 3-phosphate dehydrogenase	***GAPDH***	12:7	Yes (52)	***Gapdh***	6:125	Yes (188)
	*GAPDHS*	19:41	No	*Gapdhs*	7:30	No

Phosphoglycerate kinase	***PGK1***	X:77	Yes (3)	***Pgk1***	X:102	Yes (11)

Phosphoglycerate mutase	***PGAM1***	10:99	Yes (21)	***Pgam1***	19:42	Yes (12)
	*PGAM2*	07:44	No	*Pgam2*	11:6	No
	*PGAM5*	12:132	No	***Pgam5***	5:111	Yes (1)

Enolase	***ENO1***	01:9	Yes (4)	***Eno1***	4:149	Yes (27)
	*ENO2*	12:7	No	*Eno2*	6:125	No
	*ENO3*	17:5	No	*Eno3*	11:70	No
	*ENO4*	10:119	No	*Eno4*	19:59	No

Pyruvate kinase	*PKLR*	01:154	No	*Pklr*	3:89	No
	***PKM2***	15:70	Yes (6)	***Pkm2***	9:59	Yes (17)

Ensembl release 48 Dec 2007						

The **Position **column indicates chromosomal location denoted as "chromosome number:position (millions)". The **Ret. seq**. column indicates whether retroposed sequences were identified for each parent gene. The number in parentheses next to "Yes" in this column indicates the total number of retroposed sequences that match the parent gene. We identified a total of 94 retroposed sequences matching glycolytic enzymes in the human genome and 291 in the mouse genome.

▪ Retrotransposition of genes encoding glycolytic enzymes is frequent. We identified 94 matching retroposed sequences in the human genome and 291 in the mouse genome. Our analysis confirms that the mouse genome contains significantly more retroposed sequences than the human genome [45].

▪ As a rule, only one gene within each family is retroposed (bolded font in Table 1).

▪ The same orthologous gene is retroposed in the human and mouse genomes. This is always true in cases where there is more than one retroposed sequence. The two exceptions to this rule, hexokinase (*Hk*) and phosphoglycerate mutase (*Pgam*), have a single retroposed sequence in one or both species. In the human genome *HK2 *is retroposed, while *Hk1 *is retroposed in the mouse genome. There is also a single *Pgam5 *retroposed sequence in mouse in addition to multiple retroposed sequences for *Pgam1 *in both species.

▪ The location of retroposed sequences in the human (Additional file 1) and mouse (Additional file 2) genomes appears to be random. There is no region or chromosome with an overrepresentation of retroposed sequences. There is also evidence for segmental gene duplication of retrotransposed sequences.

▪ Retrotransposition events occurred independently in each lineage following the divergence of primates and rodents. Phylogenetic analysis was inconclusive in determining the strict order of retrotransposition events due to the high levels of sequence identity between retroposed sequences and parent genes. Analysis of genes flanking retroposed sequences confirmed that these events occurred independently in each species (Additional file 3).

**Figure 1:**
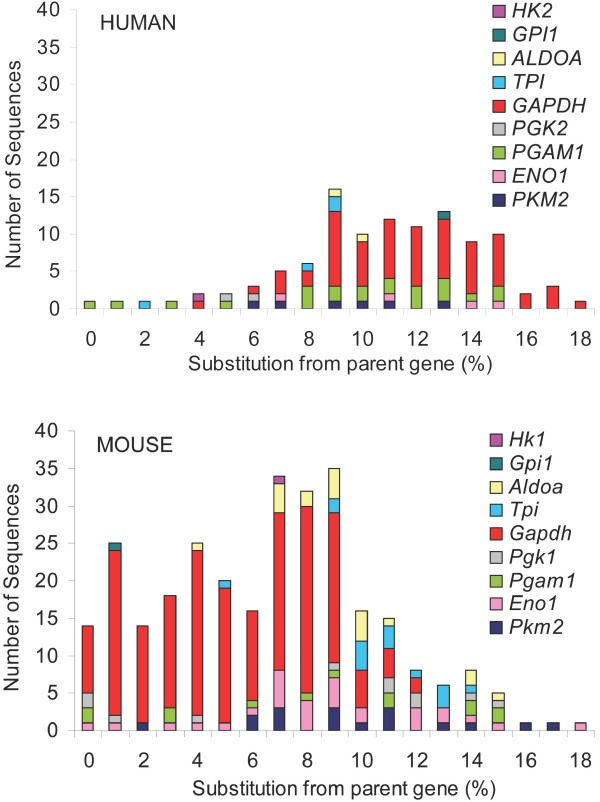
**Gene and species-specific divergence of human and mouse retroposed sequences**. Percent substitution at the nucleotide level in the entire retroposed sequences (ORFs and UTRs) compared to each parent gene. Retroposed sequences matching each enzyme are represented by a different color, as shown in the inset. Retroposed sequences for each parent gene are shown in the same order as the inset. Each retroposed sequence in represented once at the appropriate substitution level and each bar represents the number of sequences (y-axis) with the percent substitution compared to the parent gene (x-axis). Human sequences are shown on the top and mouse sequences are shown on the bottom.

▪ Human retroposed sequences derived from genes encoding glycolytic enzymes are more divergent from their parent genes than mouse retroposed sequences (Figure 1). Figure 1 groups retroposed sequences matching glycolytic enzymes by the percent nucleotide substitution in the entire sequence (ORFs and UTRs) compared to the parent gene. Human retroposed sequences are 82-100% identical to their parent genes, with a mean nucleotide identity of 89.2%. Mouse retroposed sequences have the same range of nucleotide identity, although the mean identity is 93.4%.

### 3-6% of retroposed sequences have ORFs

**Figure 2:**
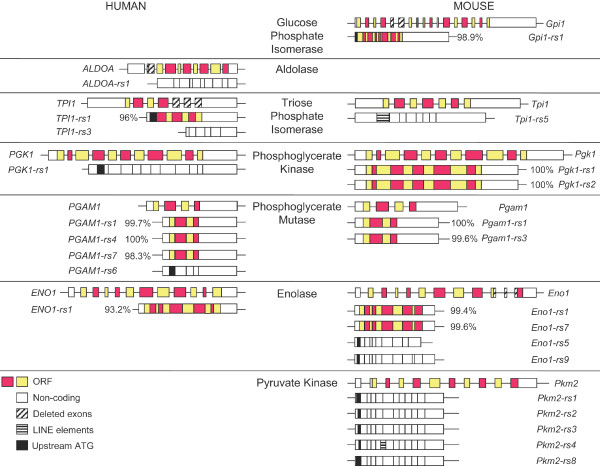
**Retroposed sequences support the expression of novel transcripts**. The structure of each parent gene is diagrammed with the coding sequence denoted by alternating yellow and red exons. Retroposed sequences with ORFs have red and yellow segments corresponding to exons in the parent gene. Upstream start codons (black exons), and/or alternatively spliced exons (diagonal lined boxes) are also shown. Sequences containing LINE elements are denoted by horizontal lines. Coding regions for retroposed sequences with ORFs were compared to their parent gene, and the percent identity at the nucleotide level is shown next to the corresponding gene structure.

6.3% of human and 3.4% of mouse retroposed sequences derived from genes encoding glycolytic enzymes contain ORFs equivalent to the full-length ORF present in the parent gene (Figure 2). This value includes the previously identified *Pgk2*, *Aldoart1*, and *Aldoart2 *retrogenes. In this study we identified five new retroposed sequences with ORFs in the human genome and seven new retroposed sequences with ORFs in the mouse genome (Figure 2, related sequences [rs] denoted with red and yellow bars representing segments that match exons in the parent gene). Numbers next to each gene structure indicate the percent identity of the coding region in each retroposed sequence compared to the parent gene.

Three of the five human retroposed sequences with ORFs (*TPI1-rs1*, *PGAM1-rs7*, *ENO1-rs1*) showed substantial divergence from their parent genes at both the nucleotide (Figure 2) and amino acid level (Additional file 4). For example, the ORF of *PGAM1-rs7 *is only 98.3% identical at the nucleotide level and encodes 11 unique amino acid residues. The mouse sequences we identified are more similar to their parent genes (99.6% identity) that humans sequences (97.4%). Six of seven mouse retroposed sequences had ORFs with >99% nucleotide and amino acid identity to their parent genes (Figure 2). Two of these sequences, *Pgk1-rs1 *and *Pgk1-rs2*, had less than 99% nucleotide identity in the 3'UTR due to a 5-base-pair insertion that we did not detect in RT-PCR analyses of testis transcripts (data not shown). *Gpi-rs1 *was the only mouse retroposed sequence that showed less than 99% sequence identity at the nucleotide (Figure 2) and amino acid level (Additional file 5). These results indicate that several retroposed sequences matching glycolytic enzymes in both the human and mouse genomes have ORFs, supporting possible expression of these sequences.

### Detection of splice variants in the glycolytic enzyme parent genes

Analysis of retroposed sequences derived from glycolytic enzyme genes support the expression of alternative transcripts from the parent genes in both the human and mouse. Two retroposed sequences in humans (*ALDOA-rs1 *and *TPI1-rs3*) suggest alternative splicing of internal exons (represented as boxes with diagonal lines in Figure 2). For example, *TPI1-rs3 *is missing 2 full consecutive exons and part of a third, but still contains sequence that matches part of the last *TPI1 *exon. Alternative splicing is also supported by two mouse retroposed sequences (*Gpi1-rs1 *and *Eno1-rs5*). For example, *Eno1-rs5 *matches full-length *Eno1*, except for a deletion of half of exon 10, exon 11 and half of exon 12 (boxes with lines, Figure 2). The remaining 3'UTR is maintained, without the coding sequence of the last exon. However, we did not detect expression of these splice variants in published EST libraries.

### Detection of N-terminal extensions in the glycolytic enzyme parent genes

Some spermatogenic-cell specific glycolytic enzymes are modified through the addition of amino acid residues at the N-terminus, including GAPDHS, ALDOA_V2 and ALDOART1 [2,4]. Our previous analysis supported the retrotransposition of an alternative splice variant (*Aldoa_v2*) to produce a novel gene encoding an N-terminal extension (*Aldoart1*) [4]. In this study, we found that multiple mouse and human retroposed sequences have upstream start codons, supporting the expression of transcripts that encode additional glycolytic enzymes with N-terminal extensions. Three human sequences (*TPI1-rs1*, *PGK1-rs1 *and *PGAM1-rs6*) and nine mouse sequences (*Gpi1-rs1*, *Tpi1-rs5, Eno1-rs5, 9*, and *Pkm2-rs1, 2, 3, 4, 8*) contain upstream start codons (black exons, Figure 2). In most cases, comparison of the amino acid sequence in these N-terminal extensions reveal a unique origin for these extensions that is independent from the parent genes (Additional file 6), since the alignment does not show a high level of identity. Five retroposed sequences matching *Pkm2 *in mice contain N-terminal extensions. Previous studies detected a larger *Pkm2 *protein in boar and mouse sperm [8,46]. Proteomic evidence from boar sperm suggests extension of the N-terminus by at least five amino acids [8]. Our sequence analysis of *Pkm2 *retropseudogenes with upstream start codons in the mouse genome shows partial agreement with the previously identified five-amino-acid extension, but does not clearly elucidate the start codon responsible for the larger protein product detected in sperm (Additional file 6).

### Novel ORFs with divergent sequences are not expressed in human testis

**Figure 3:**
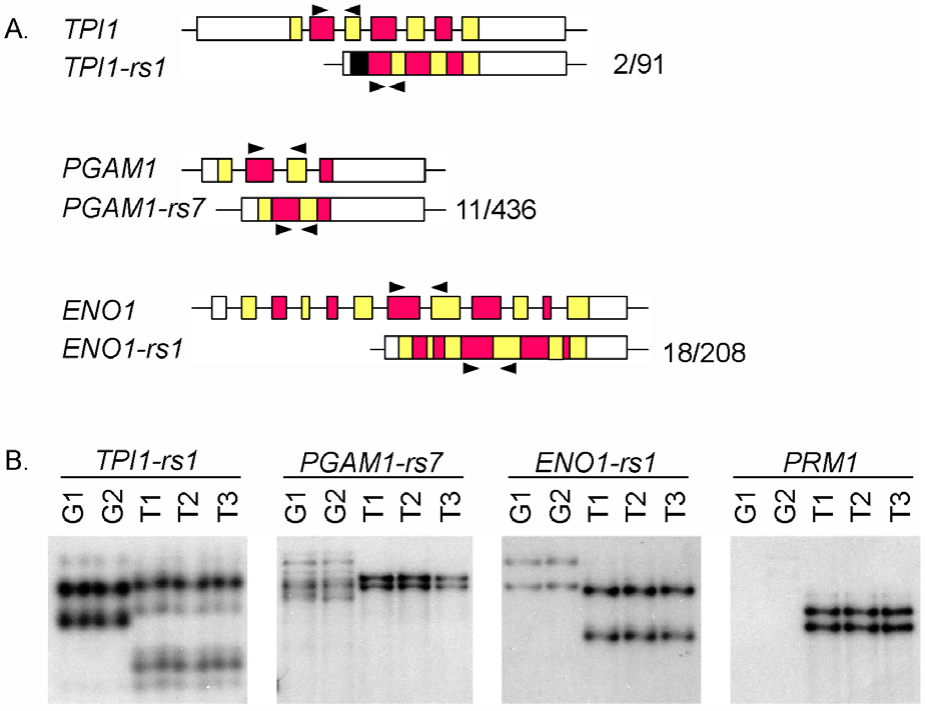
**Human ORFs with divergent sequences are not expressed in testis**. (A) Diagram of the RT-PCR approach used to distinguish expression of transcripts. Black arrows denote primer sets used to amplify both parent gene and retroposed sequences. The fraction next to each retroposed sequence shows the number of unique nucleotide residues in the amplified product. (B) *TPI1-rs1*, *PGAM1- rs7*, and *ENO1- rs1 *transcripts were not detected in pooled human testis RNA samples with RT-PCR using primers that amplify both the retroposed sequence and the parent glycolytic enzyme, followed by single-strand conformation polymorphism (SSCP) gel electrophoresis. PCR products amplified from human genomic DNA (G1 and G2; two individuals) show the expected position of transcripts from retroposed sequences. PCR products amplified from pooled testis total RNA are shown in lanes T1, T2, and T3 (triplicate cDNA preparations).

Our expression analyses in both species focused on retroposed sequences with less than 99% identity at the nucleotide level and did not include the large number of sequences derived from *GAPDH*. Due to high sequence similarity at the nucleotide level, we used Single Strand Conformation Polymorphism (SSCP) gel electrophoresis to examine potential expression of three retroposed sequences in human testis (Figure 3A). In this method RT-PCR products are denatured into two strands and separated based upon individual nucleotide differences, allowing us to distinguish between sequences with very high levels of identity. Expression of protamine 1 (*PRM1*), a spermatid-specific transcript, was detected in human cDNA preparations, confirming complete spermatogenesis in the pooled testes tissues used for RNA isolation. We used genomic DNA to identify the migration pattern of the PCR products amplified from the retroposed sequences (G1 and G2, Figure 3B). With primers specific for *TPI-rs1, PGAM1-rs7*, and *ENO1-rs1*, RT-PCR did not amplify products from human testis RNA that match the retroposed sequence fragments amplified from genomic DNA (Figure 3B). Therefore, we did not detect testis expression of the human retroposed sequences with ORFs that were analyzed in this study.

### Expression of an alternative Gpi1 transcript in mouse spermatogenic cells

**Figure 4:**
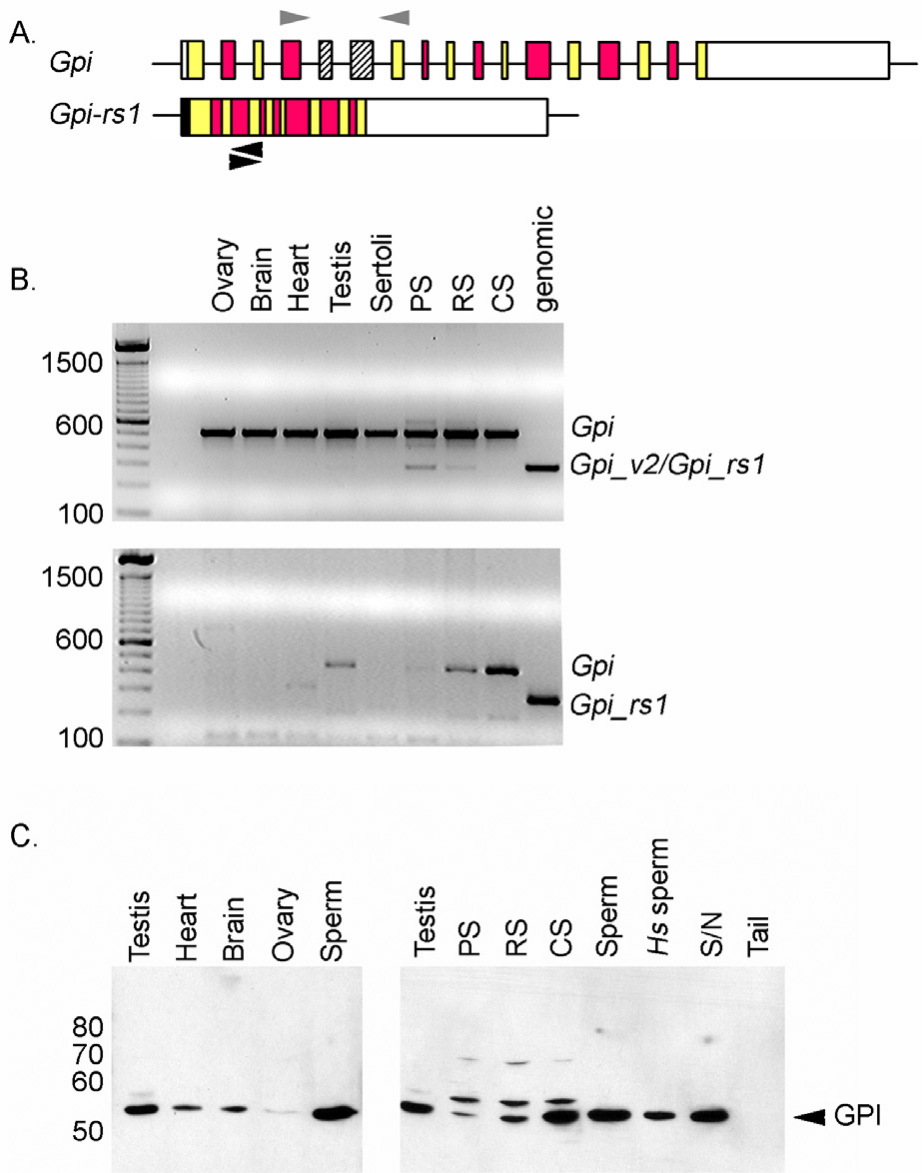
**Expression of an alternative *Gpi1 *transcript in mouse spermatogenic cells**. (A) Diagram of RT-PCR approach used to distinguish expression of *Gpi1*-related transcripts. Gray arrows denote the primer set used to differentiate transcripts containing alternatively spliced exons 5 and 6 (boxes with diagonal lines). Black arrows denote the *Gpi1-rs1*-specific primer set. (B) Transcripts from *Gpi1 *were detected in all mouse tissues and isolated testicular cells. *Gpi1-rs1 *was amplified from genomic DNA to identify the expected size of PCR products from *Gpi1 *transcripts not containing exons 5 and 6. A product of the same size was detected in isolated pachytene spermatocytes (PS) and round spermatids (RS), but not condensing spermatids (CS). This PCR fragment appears to be derived from *Gpi1_v2*, since *Gpi1_rs1-*specific primers did not amplify a product. (C) A smaller GPI1_V2 protein was not detected by western analysis using a polyclonal antibody raised against human GPI1. A larger protein product was seen in isolated testicular cell, but not in mouse or human (*Hs*) sperm. S/N fraction contains proteins solubilized from sperm tail following brief sonication and centrifugation. Tail fraction contains proteins left insoluble following sonication and centrifugation.

*Gpi1-rs1 *maintains an ORF, despite missing two internal exons (Figure 4A). The open reading frame of *Gpi1-rs1 *suggests the expression of this retroposed sequence and/or a *Gpi1 *splice variant missing exons 5 and 6 (*Gpi1_v2*). RT-PCR expression analysis in mouse tissues revealed a testis-specific transcript of glucose phosphate isomerase, representative of *Gpi1_v2 *and/or *Gpi1-rs1 *(Figure 4B). This transcript was also detected in both pachytene spermatocytes and round spermatids isolated from mouse testis, but not in later germ cells (condensing spermatids) or Sertoli cells. The same band was detected in human testis, but due to the absence of *Gpi1-rs1 *in the human genome, must represent the expression of *GPI1_V2 *(data not shown).

To distinguish between *Gpi1_v2 *and *Gpi1-rs1 *expression, we designed PCR primers to specifically detect expression of *Gpi1-rs1 *(Figure 4A). Using this approach, we did not detect a *Gpi1-rs1*-specific product (Figure 4B, bottom panel), indicating that PCR products initially detected in pachytene spermatocytes and round spermatids (Figure 4B, top panel) are most likely derived from *Gpi1_v2 *transcripts.

We detected expression of the GPI1 protein in various tissues and germ cells isolated from mouse testis (Figure 4C). GPI1 has 553 amino acids, while GPI1_V2 has 476 amino acids since it is missing sequence encoded by exons 5 and 6 (Additional file 5). GPI1-rs1 is also missing sequence encoding exons 5 and 6 but contains an N-terminal extension and is, therefore, 485 amino acids (Additional file 5). The predicted molecular weights of GPI1, GPI1_V2, and GPI1-rs1 are 62,800, 54,500 and 55,100, respectively. We detected a protein band that migrates with an apparent molecular weight of ~55,000 in all tissues analyzed. This band is assumed to be GPI1 due to its ubiquitous expression pattern. We also identified a larger immunoreactive band that was seen only in isolated spermatogenic cells (Figure 4C). This protein is not present in human or mouse sperm and is larger than the predicted molecular weights of GPI1_V2 and GPI1-rs1. We also found that glucose phosphate isomerase is soluble in the supernatant fraction following sonication of mouse sperm (S/N, Figure 4C). Since GPI1 is not found in insoluble fractions of mouse sperm, it is not tightly bound to the fibrous sheath, the cytoskeletal structure in the sperm flagellum that binds multiple glycolytic enzymes with unique N-terminal extensions [4]. Although we were unable to distinguish GPI1_V2 in our Western analysis, we identified an alternative splice variant of *Gpi1 *that is transcribed in spermatogenic cells of the mouse testis.

### Repetitive elements are overrepresented in sequence flanking retroposed sequences

The *Aldoart1 *sequence provided evidence for an alternative splice variant (*Aldoa_v2*) of aldolase A that is also expressed during spermatogenesis [4]. In our analyses of other retroposed sequences, we examined flanking sequences for evidence of alternative splicing or additional coding sequence, particularly at the N-terminus. Analysis of 1 kb sequence both upstream and downstream of all human and mouse retroposed sequences did not identify additional coding sequences. Instead, we found a significant (*p *< 0.01) increase in the number of repetitive elements, particularly LINE and LTR elements, in regions that flank retroposed sequences compared to the regions that flank the parent genes (intron-containing genes encoding the glycolytic enzymes) (Additional file 7).

**Figure 5:**
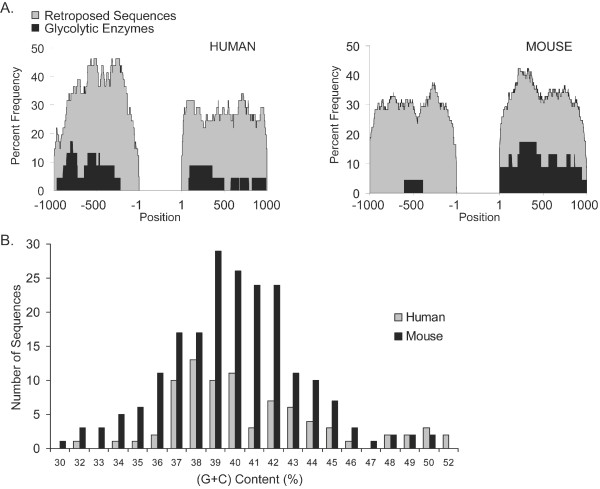
**Abundance of repetitive elements flanking retroposed sequences and (G + C) content**. (A) Diagram comparing the frequency of LINE and LTR elements in regions flanking retroposed sequences (grey) or genes encoding all glycolytic enzymes (black). (B) (G + C) content (%) of combined upstream and downstream 10 kb sequence flanking human (grey) and mouse (black) retroposed sequences.

We calculated the percent frequency of both LINE and LTR elements at each base pair within 1 kb upstream and downstream of each retroposed sequences, as compared to intron-containing parent genes encoding glycolytic enzymes. We observed an increase in LINE and LTR elements along the entire 1 kb immediately upstream or downstream of retroposed sequences (Figure 5A). Because LINE elements are found preferentially in (A + T)-rich regions of the genome [45], we expected a low (G + C) content in the flanking regions (10 kb) of retroposed sequences. Surprisingly, we found that the (G + C) content matched the (G + C) content of the entire genome for both species (Figure 5B). Therefore, these retroposed sequences and flanking repetitive elements are not preferentially located in (A + T) rich regions. We dated the repeated elements by comparing their nucleotide divergence from their respective consensus sequences, and then compared these values to the nucleotide divergence of the corresponding retroposed glycolytic sequence from the corresponding parent gene (data not shown). We find that there is no correlation.

## Discussion

We found frequent retrotransposition of one member in each gene family encoding the glycolytic enzymes. Remarkably, the orthologous gene is retroposed independently in the human and mouse genomes. Phylogenetic analyses indicate that the retroposed parent gene is not the most slowly-evolving gene in each gene family [25,31]. In support of this conclusion, phylogenetic trees for five representative glycolytic enzymes are shown in Additional file 8. Although at least one retrogene, *Pgk2*, is present in all eutherian mammals [47], most of the retroposed sequences identified in this analysis arose following the primate-rodent split. Many factors may contribute to preferential retrotransposition of a single orthologous gene in each gene family in both species.

Germline expression of the parent gene is required for retrotransposition events that can be transmitted to future generations. Retroposed parent genes in the glycolytic pathway are expressed in testicular germ cells, including *Hk1, Gpi1*, *Aldoa*, *Tpi1*, *Gapdh*, *Pgk1*, *Pgam1*, *Eno1 *and *Pkm2 *[2,4,7,9,11,48-51]. These studies and microarray analyses of isolated spermatogenic cells http://mrg.genetics.washington.edu/[52] indicate that glycolytic enzyme genes that are retroposed (Table 1) are expressed during early mitotic (spermatogonia) and/or meiotic (spermatocytes) stages of spermatogenesis in the mouse. The same microarray studies indicate that several glycolytic enzyme genes that are not retroposed are not expressed during spermatogenesis (*Hk3*, *Aldob*, *Aldoc*, *Eno2 *and *Pklr*) or are expressed only during the haploid period (*Gapdhs*). mRNA abundance and stability, as well as short length and nucleotide sequence, may contribute to preferential retrotransposition [53-55],

Along with expression of the parent gene, the machinery that facilitates retrotransposition of mRNA transcripts must also be expressed in the same developmental stage/cell type of the germline. ORF1 and ORF2, the LINE element-encoded proteins that provide RNA-binding and enzymatic activities required for retrotransposition, are expressed in testicular germ cells, including preleptotene and zygotene spermatocytes [34,36]. We found an overrepresentation of LINE and LTR repetitive elements flanking retroposed sequences derived from genes encoding glycolytic enzymes, but no evidence that both events occurred simultaneously. These retroposed sequences and flanking repetitive elements are not found in (A + T) rich regions, where LINE and LTR elements are normally found [45], suggesting a distinct mechanism for the genomic integration of these sequences compared to repetitive elements alone.

Our genomic analyses of retroposed sequences identified *Aldoart1 *and *Aldoart2*, two newly identified retrogenes in mouse [4], and may provide additional insights regarding the process of retrotransposition and the evolution of expressed retrogenes. We determined that the gene families encoding glycolytic enzymes have single members that are frequently retrotransposed, resulting in the creation of multiple gene copies in the genome. This high frequency suggests that most mammalian species, including those that are phylogenetically close, are likely to differ in the number and function of glycolytic enzymes. Some of the retroposed sequences have ORFs, due either to very recent retrotransposition events providing very little time for the accumulation of sequence divergence, or to selective pressure exerted on sequences that are expressed. Sequences with ORFs that are expressed in testicular germ cells may be acted upon by selective pressure in the context of reproductive fitness, and it is known that there is rapid evolution of genes involved in reproduction [24].

It is well known that gene duplication, including retrotransposition, provides the opportunity for the duplicated genes to diverge by mutation and eventually change or acquire new functions. In contrast with other tissues, the evolution of the glycolytic pathway in spermatogenic cells is focused on insuring the production of high levels of ATP in the sperm flagellum. There are a surprising number of glycolytic variants in mammalian sperm, and recent studies continue to uncover new enzymes and regulatory features of both glycolytic and other metabolic enzymes. For example, it is now clear that glycolysis and respiration occur in two distinct compartments of the sperm flagellum, adding complexity to the typical regulatory interactions of these metabolic pathways [56]. Furthermore, at least five glycolytic enzymes are tightly bound to the fibrous sheath, a cytoskeletal structure in the principal piece of the flagellum [4,46]. These include multiple germ cell-specific isozymes with novel N-terminal extensions that are hypothesized to play a role in localizing glycolysis in the principal piece, thereby insuring an adequate supply of ATP along the full length of the flagellum. Adaptations during the ongoing evolution of glycolysis have involved the rapid emergence of new genes by duplication and retrotransposition, the acquisition of distinct expression patterns in male germ cells, changes in the enzymatic properties, and novel structural features that facilitate compartmentalization of the glycolytic pathway in the principal piece of the sperm flagellum.

## Conclusions

We identified frequent retrotransposition in both the human and mouse genomes of a single orthologous gene in each gene family encoding glycolytic enzymes. Integration of retrotransposition events is random. Many of these retroposed sequences have ORFs and some support the expression of alternative splice variants and N-terminal extensions. These sequences are lineage-specific and many are recent, indicating that similar genomic analyses in other organisms may identify additional genes encoding glycolytic enzymes. Several of the retroposed sequences identified in this study have retained ORFs despite substantial sequence divergence, suggesting functional importance. Glycolysis is essential for sperm motility and fertilization. It is likely that selective pressure in the context of reproductive fitness contributes to the evolution of novel isozymes in this pathway.

## Methods

### Identification of gene families

The Ensembl http://www.ensembl.org Interpro Domain was used to identify the intron-containing genes for each glycolytic enzyme [57]. We used Ensembl release 48 (Dec 2007) to identify all genes, their mRNA sequences and chromosome locations. Accession numbers used for BLAST searches are:

Human: *HK1 *[ENSG00000156515] *HK2 *[ENSG00000159399] *HK3 *[ENSG00000160883], *GCK *[ENSG00000106633], *HKDC1 *[ENSG00000156510], *GPI1 *[ENSG00000105220], *PFKL *[ENSG00000141959], *PFKM *[ENSG00000152556], *PFKP *[ENSG00000067057], *ALDOA *[ENSG00000149925], *ALDOB *[ENSG00000136872], *ALDOC *[ENSG00000109107], *TPI1 *[ENSG00000111669], *GAPDH *[ENSG00000111640], *GAPDHS *[ENSG00000105679], PGK1 [ENSG00000102144], PGAM1 [ENSG00000171314], *PGAM2 *[ENSG00000164708], *PGAM5 *[ENSG00000176894], *ENO1 *[ENSG00000074800], *ENO2 *[ENSG00000111674], *ENO3 *[ENSG00000108515], *DKFZp781N1041 *[ENSG00000188316], *PKLR *[ENSG00000143627], *PKM2 *[ENSG00000067225].

Mouse: *Hk1 *[ENSMUSG00000037012], *Hk2 *[ENSMUSG00000000628], *Hk3 *[ENSMUSG00000025877], *Gck *[ENSMUSG00000041798], *Hkdc1 *[ENSMUSG00000020080], *Gpi1 *[ENSMUSG00000036427], *Pfkl *[ENSMUSG00000020277], *Pfkm *[ENSMUSG00000033065], *Pfkp *[ENSMUSG00000021196], *Aldoa *[ENSMUSG00000030695], *Aldob *[ENSMUSG00000028307], *Aldoc *[ENSMUSG00000017390], *Tpi1 *[ENSMUSG00000023456], *Gapdh *[ENSMUSG00000057666], *Gapdhs *[ENSMUSG00000061099], *Pgam1 *[ENSMUSG00000011752], *Pgam2 *[ENSMUSG00000020475], *Pgam5 *[ENSMUSG00000029500], *Eno1 *[ENSMUSG00000063524], *Eno2 *[ENSMUSG00000004267], *Eno3 *[ENSMUSG00000060600], *6430537H07Rik *[ENSMUSG00000048029], *Pklr *[ENSMUSG00000041237], *Pkm2 *[ENSMUSG00000032294].

### BLAST search for retroposed sequences

We blasted the mRNA sequence for each gene encoding a glycolytic enzyme using Ensembl BlastView in order to identify retroposed sequences in both the human and mouse genomes. We grouped BLAST hits based upon chromosome location and orientation. Multiple hits in close vicinity and with the same orientation were grouped together in a single hit that span the entire genomic sequences between and upstream/downstream of hits. Hits with less 50 base pairs were excluded. By comparing BLAST results between gene family members, we identified the parent gene for each retroposed sequence. For each retroposed sequence, we identified the parent gene by choosing matches with the longest hit and the highest percentage match. Using the BLAST results, we calculated the weighted average of the nucleotide identity of all retroposed sequences matching glycolytic enzymes. Ensembl was used to retrieve the FASTA sequence for each retroposed sequence on the appropriate strand.

### Sequence alignment

All retroposed sequences were aligned with their parent gene with Sequencher 4.8 (Gene Codes Corporation, Ann Arbor, MI). We used large gap parameters and a 60% identity threshold to align all sequences to the reference sequence (the parent gene). We examined the nucleotide sequence corresponding to the exons of the parent gene and identified insertions, deletions, and base pair substitutions. We then calculated the percent identity of the coding sequence and looked for an ORF. Amino acid sequence alignments were performed using ClustalW http://www.ebi.ac.uk/Tools/clustalw2/index.html[58].

### Tissue and cell isolations

Outbred CD-1 mice were obtained from Charles River (Raleigh, NC). All procedures involving animals were approved by the University of North Carolina at Chapel Hill Animal Care and Use Committee and conducted in accordance with the Guide for the care and Use of Laboratory Animals (Institute for Laboratory Animal Research, National Academy of Sciences).

All tissues were quick frozen in liquid nitrogen and kept at -80°C until use. Testicular germ cells were isolated using an established protocol [59]. Briefly, we purified pachytene spermatocytes, round spermatids, and condensing spermatids by unit gravity sedimentation from adult mixed germ cell suspensions [59]. Pachytene spermatocytes and round spermatids have purities >90%, while condensing spermatids have 30-40% nucleated cells and cytoplasts derived from the same cells. Testes from 17-day-old mice were used to isolate Sertoli cells, as previously described [60].

Mouse sperm was collected as previously described [4]. Briefly, each cauda epididymis was clipped and incubated for 15 minutes at 37°C in phosphate-buffered saline with protease inhibitors (PBS + PI) containing 140 mM NaCl, 10 mM phosphate buffer (pH 7.4) and Complete protease inhibitor cocktail (Roche Diagnostics, Mannheim, Germany). Cryopreserved human sperm samples from healthy donors were obtained from the Andrology Laboratory, Department of Obstetrics and Gynecology, University of North Carolina School of Medicine. These samples were washed twice with PBS to remove seminal plasma.

### RT-PCR expression analysis of newly identified retroposed sequences in mouse and human tissues and cells

Total RNA was isolated using Trizol (Invitrogen, Carlsbad, CA) from isolated testicular cells or tissues pooled from at least three mice. Adult tissues included brain, heart, ovary and testis. The Qiagen RNeasy Midi Kit (Qiagen Incorporation, Valencia, CA) was used to remove genomic DNA contamination from RNA preparations. RNA was quantified using the NanoDrop spectrophotometer (NanoDrop Technologies, Wilmington, DE). Human RNA prepared from tissues pooled from 39 individuals was purchased from Clontech (Mountainview, CA). Genomic DNA isolated from CD-1 mice and two human subjects were used as positive controls for detected of PCR-amplified retrogenes. Reverse transcription followed by gene-specific polymerase chain reaction (Superscript RT II, Invitrogen, Carlsbad, CA; Taq DNA polymerase, New England BioLabs, Ipswich, MA) was used to amplify transcripts from total RNA samples.

Two primer pairs were designed to detect expression of *Gpi1*-related transcripts in mouse RNA samples. The first primer pair distinguishes between transcripts that contain alternatively spliced exons 5 and 6 (Figure 4A): *Gpi1*F in exon 4 (5'GAGGTGAACAGGGTTCTGGA3'), *Gpi1*R in exon 11 (5'GCTCGAAGTGGTCAAAACC3'). The expected product sizes are as follows: *Gpi1*, 520 base pairs; *Gpi1_v2/Gpi1-rs1*, 288 base pairs. The second primer pair is specific for *Gpi1*-*rs1*F in exon 4 (5'ATCAAGGTGGTCGGG3'), *Gpi1-rs1 *R in exon 10 (5'CAATGGAAGGTCCAG3'). We also included a negative control with no reverse transcriptase as a control for genomic DNA contamination. All PCR products were resolved by 2% agarose gel electrophoresis and visualized by ethidium bromide staining using UV detection.

To detect expression of human retroposed sequences, primers were designed to amplify and incorporate α-[^32^P]-dCTP into the same size product from the parent gene and the retroposed sequences of interest: *Tpi1/Tpi1-rs1, Pgam1/Pgam1-rs1*, and *Eno1/Eno1-rs1*. The forward primer sequence for *Tpi1/Tpi1-rs1 *was 5'CCGACACCGAGGTGGTTT3' and the reverse primer sequence was 5'GTTCTGCGCAGCCACAGCAA3'. The forward primer sequence for *Pgam1/Pgam1-rs1 *was 5'GCAGACCTCACAGAAGATCAG3' and the reverse primer sequence was 5'ACAGATGTGGTCAGTGTGACAT3'. The forward primer sequence for *Eno1/Eno1-rs1 *was 5'TTGGGAAAGCTGGCTACACT3' and the reverse primer sequence was 5' CCAGTCATCCTGGTCAAAGG 3'. Arrows in Figure 3A denote the location of these primers in each gene.

As a positive control to confirm proper spermatogenesis in human testis samples, we detected expression of protamine 1 (*Prm1*) in RNA samples. We also included a negative control with no reverse transcriptase as a control for genomic DNA contamination. The forward primer sequence for *Prm1 *was 5'TCACAGGTTGGCTGGCTC3'and the reverse primer sequence was 5'CATTGTTCCTTAGCAGGCTCC3' [61]. Following PCR amplification with both primer sets, the products were resolved by Single Strand Conformation Polymorphism (SSCP) electrophoresis using MDE gel solution (Cambrex, East Rutherford, NJ) at 0.5 W for 19 hours. Genomic DNA was used as a control template in parallel PCR reactions to confirm the expected electrophoretic pattern of the retroposed sequences. Gels were exposed to Super RX X-ray film (Fujifilm, Tokyo, Japan) using intensifying screens to detect incorporation of α-[^32^P]-dCTP into amplified products.

### Western analysis of GPI1-related proteins

Lysis buffer (2% SDS, 100 mM DTT, 125 mM Tris pH 6.8, 18% glycerol) was used to extract proteins from tissues or isolated cells. Samples were centrifuged at 16,000 × *g *for 10 min at 4°C following homogenization. Protein concentrations were determined using the micro-BCA assay (Pierce Biotechnology, Rockford, IL). SDS polyacrylamide gel electrophoresis (SDS-PAGE) on 7.5% polyacrylamide gels was used to separate samples with equal protein amounts, followed by electrophoretic transfer to Immobilon-P PVDF (polyvinylidene fluoride) membranes (Millipore Corp, Bedford, MA). Equal protein loading was confirmed by Coomassie blue R250 staining (0.1% Coomassie blue R250 in 45% methanol, 10% acetic acid). Membranes were destained, rinsed with TBS-T (140 mM NaCl, 3 mM KCl, 0.05% Tween-20, 25 mM Tris-HCl, pH 7.4) and incubated in blocking buffer (5% nonfat dry milk in TBS-T) overnight at 4°C. Antibody incubations were performed at room temperature in blocking buffer. Membranes were incubated with a 1:500 dilution of a polyclonal antibody raised against a recombinant human glucose phosphate isomerase protein fragment (Strategic Diagnostic Incorporation, Newark, DE) for 2 hours. Membranes were then incubated for 45 min at room temperature with secondary antibody (affinity-purified horseradish peroxidase-conjugated rabbit anti-goat IgG, KPL, Gaithersburg, MD) diluted 1:10,000. Following antibody incubations, membranes were rinsed for 5 minutes with TBS-T. Immunoreactive proteins were detected by enhanced chemiluminescence using the SuperSignal West Pico substrate (Pierce Biotechnology, Rockford, IL) and HyBlot CL autoradiography film (Denville Scientific, Metuchen, NJ).

### Repetitive Element Analysis

Galaxy http://galaxy.psu.edu was used to obtain both 1 kb and 10 kb FASTA format sequence flanking retroposed sequences and genes encoding glycolytic enzymes [62]. We analyzed FASTA format sequence for all retroposed sequences, 1 kb flanking retroposed sequences and 1 kb flanking genes encoding glycolytic enzymes for the presence of repetitive elements using Repeatmasker http://www.repeatmasker.org. We calculated the percent frequency of repetitive elements (LINE, LTR, and SINE) in each base pair within 1 kb upstream or downstream of retroposed sequences or genes encoding glycolytic enzymes. Chi-square values were calculated using a contingency table comparing mouse and human sequences versus glycolytic enzymes and retroposed sequences for each repetitive element. (G + C) content was calculated using the eMBOSS geecee program http://inn-temp.weizmann.ac.il/cgi-bin/emboss/geecee[63].

### BLAST search for extensions

Repeatmasker http://www.repeatmasker.org was used to generate sequence with the repetitive elements masked (represented by "n") [64]. We repeatmasked the 1 kb sequence flanking all retroposed sequences and used Ensembl BLAST to compare this sequence to the mouse or human genome. We looked for matches with genomic locations close to either the parent gene or other retroposed sequences, indicative of a sequence extension at the end of the gene.

### Dating retroposed sequences

We dated the repeated elements by comparing their nucleotide divergence from their respective consensus and then compared these values to the nucleotide divergence of the corresponding retroposed glycolytic sequence to the corresponding parent gene. In addition, we determined whether retroposed sequences are located at homologous position of the human and mouse genome by determining the position of the flanking genes in the appropriate species. We then found the position of the homologous genes in the others species using comparative maps http://www.ncbi.nlm.nih.gov/projects/homology/maps/. Finally, to determine the evolutionary history of genes within each gene family and their rate of divergence we aligned the coding sequence using ClustalW http://www.ebi.ac.uk/Tools/clustalw2/index.html[58] and constructed a distance tree using the Neighbor Joining method from the PHYLIP package http://evolution.genetics.washington.edu/phylip/.

## List of Abbreviations

Hk: Hexokinase; Gpi1: Glucose phosphate isomerise; Pfk: Phosphofructokinase; Aldo: Aldolase; Tpi1: Triose phosphate isomerise; Gapdh: Glyceraldehyde phosphate dehydrogenase; Pgk: Phosphoglycerate kinase; Pgam: Phosphoglycerate mutase; Eno: Enolase; Pk: Pyruvate kinase; Ldh: Lactate dehydrogenase; LINE: Long interspersed nucleotide element; LTR: Long terminal repeats; SINE: Short interspersed nucleotide element; SSCP: Single strand conformation polymorphism electrophoresis; ATP: Adenosine triphosphate; RT-PCR: Reverse transcription polymerase chain reaction; ORFs: open reading frames.

## Authors' contributions

SAV developed and performed experiments, analyzed data, and drafted the manuscript. FPMV and DAO conceived of the study, participated in experimental design, and edited the manuscript. All authors read and approved the final manuscript.

## Supplementary Material

Additional file 1 **Human retroposed sequences matching genes encoding glycolytic enzymes**. This table indicates the gene name for each retroposed sequence in the human genome, along with the chromosome position and strand. FL CDS refers to those sequences containing full-length coding sequence (Y) or only untranslated sequence (UTR), regardless of whether the sequences are in frame. In some cases, multiple retroposed sequences from the same parent gene are located at adjacent chromosome positions. For example *ENO1-rs2 *and *ENO1-rs3 *are located less than 2 kb apart on chromosome 15. Click here for file

Additional file 2 **Mouse retroposed sequences matching genes encoding glycolytic enzymes**. This table indicates the gene name for each retroposed sequence in the mouse genome, along with the chromosome position and strand. FL CDS refers to those sequences containing full-length coding sequence (Y) or only untranslated sequence (UTR), regardless of whether the sequences are in frame. In some cases, multiple retroposed sequences from the same parent gene are located at adjacent chromosome positions. For example: *Eno1-rs15 and Eno1-rs23 *are located less than 1 kb apart on chromosome 3 m and *Pkm2-rs2 *and *Pkm2-rs3 *are located 150 kb apart on chromosome 2. Click here for file

Additional file 3 **The position of genes flanking retroposed sequences in the mouse and human genome**. The table identifies the genes flanking each of the retroposed sequences derived from genes encoding glycolytic enzymes. For each retroposed sequenced we determined the position of the flanking genes in the appropriate species and determined the position of the homologous genes in the others species using well established comparative maps http://www.ncbi.nlm.nih.gov/projects/homology/maps/. Click here for file

Additional file 4 **Amino acid alignment of retroposed sequences in the human genome that maintain ORFs**. Asterisks (*) denote identical residues. Methionine residues are highlighted in grey boxes, residues marked as "X" in a black box denote stop codons, and dashes denote deleted codons. Click here for file

Additional file 5 **Amino acid alignment of GPI1-related sequences in the mouse genome that maintain ORFs (GPI1-rs1)**. Asterisks (*) denote identical residues. Methionine residues are highlighted in grey boxes, dashes denote deleted codons and the stop codon is marked as "X" in a black box. Click here for file

Additional file 6 **Amino acid alignments are shown for retroposed sequences containing upstream start codons**. (A) Amino acid sequence alignment comparing upstream extensions of human retroposed sequences to their parent glycolytic enzymes. (B) Amino acid sequence alignment of mouse retroposed sequences with upstream start codons and their parent glycolytic enzymes. Asterisks (*) denote identical residues. Methionine residues are highlighted in grey boxes, residues marked as "X" in a black box denote stop codons, and dashes indicate deleted codons. Pkm2-*Ss *sequence is from [8]. *Hs (Homo sapiens), Bt (Bos taurus), Ss (Su scrofa), Mm (Mus musculus)*. Click here for file

Additional file 7 **Percent frequency of repetitive elements flanking retroposed sequences and genes encoding glycolytic enzymes in the (A) human and (B) mouse genomes**. Black bars represent the percent frequency of SINE, LINE and LTR elements flanking all intron-containing parent genes that encode glycolytic enzymes. Gray bars denote the percent frequency of SINE, LINE and LTR elements both upstream and downstream of retroposed sequences derived from these parent genes. White bars represent the genome average frequency of these elements, as determined by Waterston *et al*., 2002 [45]. Click here for file

Additional file 8 **Retroposed and non-retroposed genes evolve at similar rates within each gene family**. The figure shows the phylogenetic trees for five gene families (glyceraldehyde 3-phosphate dehydrogenase, pyruvate kinase, aldolase, phosphoglycerate mutase and hexokinase). h, denotes human genes; m, denote mouses genes. Black lines are used in branches for which we did not find evidence of retrotransposition. Red lines represent branches with evidence of retrotransposition. Numbers denote the average branch length since the primate/rodent split. Click here for file
