# Supplementary material for: Frequent and recent retrotransposition of orthologous genes plays a role in the evolution of sperm glycolytic enzymes
Source: BMC Genomics. 2010 May 6;11:285. doi: 10.1186/1471-2164-11-285 (PMC2881024; doi:10.1186/1471-2164-11-285)
Supplement: Additional file 1 — Human retroposed sequences matching genes encoding glycolytic enzymes. This table indicates the gene name for each retroposed sequence in the human genome, along with the chromosome position and strand. FL CDS refers to those sequences containing full-length coding sequence (Y) or only untranslated sequence (UTR), regardless of whether the sequences are in frame. In some cases, multiple retroposed sequences from the same parent gene are located at adjacent chromosome positions. For example ENO1-rs2 and ENO1-rs3 are located less than 2 kb apart on chromosome 15. [file 1471-2164-11-285-S1.PDF]

Human retroposed sequences matching genes encoding glycolytic enzymes

Parent Gene	Gene name	Chr	Start	End	Strand	FL CDS
HK2	*HK2-rs1*	X	79711597	79717193	-	Y
GPI1	*GPI1-rs1*	3	53014814	53015169	-	
ALDOA	*ALDOA-rs1*	10	127345239	127346612	-	Y
	ALDOA-rs2	3	52202205	52204044	+	
TPI1	*TPI1-rs1*	1	76938028	76939260	+	Y
	TPI1-rs2	7	128483271	128484502	+	Y
	TPI1-rs3	4	48713051	48713961	-	Y
	TPI1-rs4	6	116466583	116467789	+	Y
PGK1	*PGK1-rs1*	X	67206648	67208343	-	Y
	PGK1-rs2	19	12531320	12533092	-	Y
PGAM1	*PGAM1-rs1*	12	102948637	102950343	+	Y
	PGAM1-rs2	15	94784888	94786599	-	Y
	PGAM1-rs3	3	9365078	9366726	-	Y
	PGAM1-rs4	12	92557837	92559523	-	Y
	PGAM1-rs5	12	94590196	94591866	+	Y
	PGAM1-rs6	6	73821525	73823177	+	Y
	PGAM1-rs7	X	77110148	77111803	-	Y
	PGAM1-rs8	11	91738423	91740066	-	Y
	PGAM1-rs9	X	46390044	46391709	-	
	PGAM1-rs10	9	35932321	35933832	-	
	PGAM1-rs11	20	11549802	11551012	+	
	PGAM1-rs12	X	54715275	54717274	+	
	PGAM1-rs13	11	64698204	64699538	-	
	PGAM1-rs14	4	116903998	116905323	-	
	PGAM1-rs15	13	59794725	59795890	+	
	PGAM1-rs16	12	46570073	46570979	-	UTR
	PGAM1-rs17	2	23948642	23949404	-	
	PGAM1-rs18	1	10041994	10042676	+	
	PGAM1-rs19	5	57492909	57493336	+	
	PGAM1-rs20	8	108728816	108729137	+	
	PGAM1-rs21	7	7410252	7410423	-	UTR
ENO1	*ENO1-rs1*	1	234713027	234714826	+	Y
	ENO1-rs2	15	90765047	90765198	-	
	ENO1-rs3	15	90766554	90766652	-	
	ENO1-rs4	9	22053949	22054012	-	UTR
PKM2	*PKM2-rs1*	1	114880051	114882605	+	Y
	PKM2-rs2	6	5917597	5919858	+	Y
	PKM2-rs3	X	65634319	65636611	+	Y
	PKM2-rs4	8	76451507	76452423	-	
	PKM2-rs5	5	8779377	8780055	+	
	PKM2-rs6	6	86426308	86426934	-	
