# Supplementary material for: Frequent and recent retrotransposition of orthologous genes plays a role in the evolution of sperm glycolytic enzymes
Source: BMC Genomics. 2010 May 6;11:285. doi: 10.1186/1471-2164-11-285 (PMC2881024; doi:10.1186/1471-2164-11-285)
Supplement: Additional file 2 — Mouse retroposed sequences matching genes encoding glycolytic enzymes. This table indicates the gene name for each retroposed sequence in the mouse genome, along with the chromosome position and strand. FL CDS refers to those sequences containing full-length coding sequence (Y) or only untranslated sequence (UTR), regardless of whether the sequences are in frame. In some cases, multiple retroposed sequences from the same parent gene are located at adjacent chromosome positions. For example: Eno1-rs15 and Eno1-rs23 are located less than 1 kb apart on chromosome 3 m and Pkm2-rs2 and Pkm2-rs3 are located 150 kb apart on chromosome 2. [file 1471-2164-11-285-S2.PDF]

Mouse retroposed sequences matching genes encoding glycolytic enzymes

Parent Gene	Gene name	Chr	Start	End	Strand	FL CDS
Hk1	*Hk1-rs1*	18	29504342	29504735	+	UTR
Gpi1	*Gpi1-rs1*	8	52202205	52204044	+	Y
Tpi1	*Tpi1-rs1*	14	17779166	17780521	+	Y
	Tpi1-rs2	10	44309731	44312928	+	
	Tpi1-rs3	9	31755930	31757215	+	Y
	Tpi1-rs4	18	20706529	20707859	-	Y
	Tpi1-rs5	10	18821352	18822679	+	Y
	Tpi1-rs6	3	65932328	65933674	-	Y
	Tpi1-rs7	13	25547615	25548396	+	
	Tpi1-rs8	5	83621602	83622074	-	UTR
	Tpi1-rs9	2	9191857	9192103	-	
	Tpi1-rs10	7	54502936	54503214	+	UTR
	Tpi1-rs11	7	54777639	54777893	+	UTR
	Tpi1-rs12	7	55039236	55039446	+	UTR
	Tpi1-rs13	7	54608077	54608280	+	UTR
	Tpi1-rs14	7	55246555	55246649	+	UTR
	Tpi1-rs15	7	54503234	54503328	+	UTR
Pgk1	*Pgk1-rs1*	12	10905372	10907119	-	Y
	Pgk1-rs2	3	57906493	57908218	-	Y
	Pgk1-rs3	6	29911075	29912501	+	
	Pgk1-rs4	3	18464493	18465979	+	Y
	Pgk1-rs5	5	85295224	85295820	-	
	Pgk1-rs6	18	6760085	6760954	-	
	Pgk1-rs7	12	94954868	94955589	-	
	Pgk1-rs8	16	81909252	81909959	+	
	Pgk1-rs9	5	85290883	85291246	-	UTR
	Pgk1-rs10	14	86629605	86629757	+	UTR
	Pgk1-rs11	14	86629502	86629607	-	
Pgam1	*Pgam1-rs1*	7	28253905	28255638	-	Y
	Pgam1-rs2	11	21582397	21584105	-	Y
	Pgam1-rs3	3	11013514	11015192	-	Y
	Pgam1-rs4	11	79710680	79712360	+	Y
	Pgam1-rs5	18	80359524	80361179	+	
	Pgam1-rs6	15	59760341	59761759	-	
	Pgam1-rs7	9	50023246	50024730	+	
	Pgam1-rs8	1	9429117	9430492	+	
	Pgam1-rs9	X	136493319	136494149	+	UTR
	Pgam1-rs10	15	24059372	24059829	-	
	Pgam1-rs11	1	23984587	23984906	-	
	Pgam1-rs12	9	123442121	123442509	+	UTR
Pgam5	*Pgam5-rs1*	12	92720490	92723471	+	Y
Eno1	*Eno1-rs1*	18	48206301	48208032	+	Y
	Eno1-rs2	14	15279830	15281567	-	Y
	Eno1-rs3	3	130631969	130633696	-	Y
	Eno1-rs4	17	16918472	16920172	+	Y
	Eno1-rs5	13	88686724	88688161	+	Y
	Eno1-rs6	18	48227671	48229782	-	Y
	Eno1-rs7	2	80677252	80678919	+	Y
	Eno1-rs8	X	126935186	126936782	-	Y
	Eno1-rs9	1	43842756	43844341	+	
	Eno1-rs10	2	100247310	100248846	-	Y
	Eno1-rs11	17	91191800	91193200	-	
	Eno1-rs12	4	64517715	64519081	-	
	Eno1-rs13	X	104474124	104475051	-	
	Eno1-rs14	10	18981708	18982788	+	
	Eno1-rs15	3	60573575	60574378	-	
	Eno1-rs16	10	68153941	68154564	+	

	Eno1-rs17	7	4902248	4902901	-	
	Eno1-rs18	2	83378374	83378827	-	
	Eno1-rs19	12	64765950	64766281	+	
	Eno1-rs20	1	165944207	165944509	-	UTR
	Eno1-rs21	1	193075309	193075608	+	
	Eno1-rs22	8	51205678	51205966	-	UTR
	Eno1-rs23	3	60574381	60574576	+	
	Eno1-rs24	14	53128289	53128461	-	
	Eno1-rs25	10	10895171	10895287	-	UTR
	Eno1-rs26	3	13489047	13489153	+	UTR
	Eno1-rs27	16	82107093	82107155	-	
Pkm2	*Pkm2-rs1*	12	31702976	31705204	+	Y
	Pkm2-rs2	2	100299806	100301960	-	Y
	Pkm2-rs3	2	100466315	100468469	-	Y
	Pkm2-rs4	9	97108961	97111315	-	Y
	Pkm2-rs5	X	111135597	111137719	+	Y
	Pkm2-rs6	X	8499059	8501032	-	
	Pkm2-rs7	5	22230028	22232077	-	Y
	Pkm2-rs8	15	69893125	69895146	-	Y
	Pkm2-rs9	18	21650759	21652605	-	Y
	Pkm2-rs10	13	13987730	13988873	-	
	Pkm2-rs11	X	159282193	159283455	-	
	Pkm2-rs12	12	88056199	88056992	+	
	Pkm2-rs13	17	93117277	93118318	-	
	Pkm2-rs14	3	43567795	43568688	-	
	Pkm2-rs15	16	54827438	54827861	-	
	Pkm2-rs16	13	14002561	14002728	-	
	Pkm2-rs17	19	54333380	54333485	-	UTR
