# Supplementary material for: Frequent and recent retrotransposition of orthologous genes plays a role in the evolution of sperm glycolytic enzymes
Source: BMC Genomics. 2010 May 6;11:285. doi: 10.1186/1471-2164-11-285 (PMC2881024; doi:10.1186/1471-2164-11-285)
Supplement: Additional file 3 — The position of genes flanking retroposed sequences in the mouse and human genome. The table identifies the genes flanking each of the retroposed sequences derived from genes encoding glycolytic enzymes. For each retroposed sequenced we determined the position of the flanking genes in the appropriate species and determined the position of the homologous genes in the others species using well established comparative maps http://www.ncbi.nlm.nih.gov/projects/homology/maps/. [file 1471-2164-11-285-S3.DOC]

The position of genes flanking retroposed sequences in the mouse and human genome

HUMAN MOUSE Locus Chr Mb Locus Chr Mb

*NMNAT1* 1 10,003,486 *Nmnat1* 4 148,842,896

*PGAM1-rs18* 1 10,041,994 - - -

*RBP7* 1 10,057,255 *Rbp7* 4 148,823,810

*ST6GALNAC3* 1 76,540,404 *St6galnac3* 3 152,865,473

*TPI1-rs1* 1 76,938,028 - - -

*ST6GALNAC5* 1 77,333,186 *St6galnac5* 3 152,482,850

*SYT6* 1 114,631,914 *STY6* 3 103,379,204

*PKM2-rs1* 1 114,880,051 - - -

TRIM33 1 114,935,399 *TRIM33* 3 103,083,216

*C1orf31* 1 234,509,429 *1810063B05Rik* 8 128,946,428

*ENO1-rs1* 1 234,713,027 - - -

*IRF2BP2* 1 234,740,015 *Irf2bp2* 8 129,112,194

*KCNS3* 2 18,059,945 *Kcns3* 12 11,097,008

- - - *Pgk1-rs1* 12 10,905,372

*RDH14* 2 18,735,989 *Rhd14* 12 10,397,586

*KLHL29* 2 23,608,557 *Klhl29* 12 5,084,275

*PGAM1-rs17* 2 23,948,642 - - -

*ATAD2B* 2 23,971,534 *Atad2b* 12 4,924,159

*OTX1* 2 63,277,965 *Otx1* 11 21,894,767

- - - *Pgam1-rs2* 11 21,582,397

*C2orf86* 2 63,348,518 *Cv249152* 11 21,472,284

*DUSP19* 2 183,943,287 *Dusp19* 2 80,457,371

- - - *Eno1-rs7* 2 80,677,252

*ZNF804A* 2 185,463,093 *Zfp804a* 2 81,893,815

*SRGAP3* 3 9,022,278 *Srgap3* 6 112,672,782

*PGAM1-rs3* 3 9,365,078 - - -

*THUMPD3* 3 9,404,717 *Thumpd3* 6 112,996,321

*THRB* 3 24,158,644 *Thrb* 14 18,814,089

- - - *Tpi1-rs1* 14 17,779,166

*RARB* 3 25,469,754 *Rarb* 14 17,263,353

*SACM1L* 3 45,730,754 *Sacm1l* 9 123,435,000

- - - *Pgam1-rs12* 9 123,442,121

*SLC6A20* 3 45,796,941 *Xtrp3s1* 9 123,545,240

*WDR51A* 3 52,109,270 *Wdr51a* 9 106,183,732

*ALDOA-rs2* 3 52,202,205 - - -

*ALAS1* 3 52,232,116 *Alas1* 9 106,136,258

*SFMBT1* 3 52,938,627 *Sfmbt1* 14 31,579,186

*GPI1-rs1* 3 53,014,814 - - -

*RFT1* 3 52,938,627 *Sfmbt1* 14 31,579,186

*PFN2* 3 149,682,691 *Pfn2* 3 57,645,817

- - - *Pgk1-rs2* 3 57,906,493

*TSC22D2* 3 150,126,788 *Tsc22d2* 3 58,219,611

*VEPH1* 3 156,978,698 *Veph1* 3 65,860,557

- - - *Tpi1-rs6* 3 65,932,328

*PTX3* 3 157,154,580 *Ptx3* 3 66,023,809

*FRYL* 4 48,499,380 *Fryl* 5 73,411,430

*TPI1-rs3* 4 48,713,051 - - -

*OCIAD1* 4 48,833,015 *Ociad1* 5 73,684,063

*LPHN3* 4 62,362,839 *Lphn3* 5 81,450,618

- - - *Tpi1-rs8* 5 83,621,602

*SRD582L2* 4 65,144,177 *Srd582l2* 5 83,707,173

*EPHA5* 4 66,185,281 *Epha5* 5 84,486,816

- - - *Pgk1-rs9* 5 85,290,883

- - - *Pgk1-rs5* 5 85,295,224

*CENPC1* 4 68,337,989 *Cenpc1* 5 86,441,049

*LEF1* 4 108,968,701 *Lef1* 3 130,813,389

- - - *Eno1-rs3* 3 130,631,969

*OSTC* 4 129,571,741 *2310008M10Rik* 3 130,398,837

*NDST4* 4 115,748,931 *Ndst4* 3 125,140,598

*PGAM1-rs14* 4 116,903,998 - - -

*TRAM1L1* 4 118,004,710 *Tram1l1* 3 124,023,955

*AGA* 4 178,351,924 *Aga* 8 54,597,080

- - - *Gpi1-rs1* 8 52,202,205

*ODZ3* 4 183,245,137 *Odz3* 8 49,313,038

*BASP1* 5 17,217,750 *Basp1* 15 25,293,984

- - - *Pgam1-rs10* 15 24,059,372

*CDH12* 5 21,750,973 *Cdh1* 15 21,041,207

*ACTBL2* 5 56,775,843 *4732495G21Rik* 13 112,045,221

*PGAM1-rs19* 5 57,492,909 - - -

*PLK2* 5 57,749,809 *Plk2* 13 111,185,270

*EDIL3* 5 83,238,126 *Edil3* 13 88,961,118

- - - *Eno1-rs5* 13 88,686,724

*RASA1* 5 86,564,151 *Rasa1* 13 85,355,631

*RIOK2* 5 96,498,641 *Riok2* 17 17,506,500

- - - *Eno1-rs4* 17 16,918,472

*RGMB* 5 98,104,999 *Rgmb* 17 15,943,217

*SEMA6A* 5 115,779,251 *Sema6a* 18 47,395,252

- - - *Eno1-rs6* 18 48227671

- - - *Eno1-rs1* 18 48206301

*DTWD2* 5 118,172,579 *Dtwd2*  18 49,856,468

*FARS2* 6 5,261,584 *Fars2* 13 36,209,280

*PKM2-rs2* 6 5,917,597 - - -

*NRM1* 6 5,998,232 *Nrm1* 13 36,817,494

*CAGE1* 6 7,326,887 *Cage1* 13 38,097,924

*PGAM1-rs6* 6 73,821,525 - - -

*RIOK1* 6 7,390,062 *Riok1* 13 38,129,164

*SLC35B3* 6 8,413,301 *Slc35b3* 13 39,024,009

*PKM2-rs6* 6 86,426,308 - - -

*TFAP2A* 6 10,396,916 *Tcfap2a* 13 40,812,101

*PRL* 6 22,287,480 *Prl* 13 27,149,445

- - - *Tpi1-rs7* 13 25,547,615

*NRSN1* 6 24,126,414 *Nrsn1* 13 25,343,909

*SMAP1* 6 71,377,479 *Smap1* 1 23,852,466

- - - *Pgam1-rs11* 1 23,984,587

*C6orf57* 6 71,276,625 *1110058L19Rik* 1 24,002,785

*PREP* 6 105,725,506 *Prep* 10 44,787,020

- - - *Tpi1-rs2* 10 44,309,731

*PRDM1* 6 106,534,195 *Prdm1* 10 44,156,983

*OLIG3* 6 137,813,336 *Olig3* 10 19,076,345

- - - *Tpi1-rs5* 10 18,821,352

*TNFAIP3* 6 138,188,581 *Tnfaip3* 10 18,720,722

*COL10A1* 6 116,440,085 *Col10a1* 10 34,109,641

*TPI1-rs4* 6 116,466,583 - - -

*TSPYL4* 6 116,571,127 *Tspyl4* 10 34,017,227

*COL28A1* 7 7,398,244 *Col28a1* 6 7,947,808

*PGAM1-rs21* 7 7,410,252 - - -

*MIOS* 7 7,606,616 *BC020002* 6 8,159,229

*FLNC* 7 128,470,483 *Flnc* 6 29,383,153

*TPI1-rs2* 7 128,483,271 - - -

*ATP6V1F* 7 128,502,898 *Atp6v1f* 6 29,417,783

*FAM40B* 7 129,074,274 *D330017J20Rik* 6 29,867,013

- - - *Pgk1-rs3* 6 29,911,075

*LOC100287482* 7 129,142,320 *1700023L04Rik* 6 29,930,731

*SPAG11B* 8 7,305,276 *EG546038* 8 19,140,759

*PKM2-rs4* 8 76,451,507 - - -

*DEFB106A* 8 7,682,694 *Defb15* 8 23,040,285

*SNTG1* 8 51,306,754 *Sntg1* 1 8,351,556

- - - *Pgam1-rs8* 1 9429117

*MYBL1* 8 67,474,410 *Mybl1* 1 9,658,918

*CYP7B1* 8 65,508,529 *Cyp7b1* 3 17,971,950

- - - *Pgk1-rs4* 3 18,464,493

*ARMC1* 8 66,515,064 *Armc1* 3 19,032,144

*SNX16* 8 82,711,818 *Snx16* 3 10,418,147

- - - *Pgam1-rs3* 3 11,013,514

*RALYL* 8 85,095,453 *0710005M24Rik* 3 13,471,682

*ANGPT1* 8 108,261,710 *Angpt1* 15 42,256,269

*PGAM1-rs20* 8 108,728,816 - - -

*RSPO2* 8 108,911,544 *Rspo2* 15 42,852,341

*CDKN2B* 9 22,002,902 *Cdkn2b* 4 88,952,229

*ENO1-rs4* 9 22,053,949 - - -

*DMRTA1* 9 22,446,840 *Dmrta1* 4 89,354,889

*OR13J1* 9 35,869,460 *Olfr71* 4 43,718,500

*PGAM1-rs10* 9 35,932,321 - - -

*OR2S2* 9 35,957,105 *Olfr159* 4 43,782,922

*GATA3* 10 8,096,667 *Gata3* 2 9,778,705

- - - *Tpi1-rs9* 2 9,191,857

*CUGBP2* 10 11,047,259 *Cugbp2* 2 6,460,740

*RAB18* 10 27,793,249 *Rab18* 18 6,765,203

- - - *Pgk1-rs6* 18 6,760,085

*EPC1* 10 32,557,859 *Epc1* 18 6,435,949

*C10orf122* 10 127,344,263 *4930404H21Rik* 7 140,778,707

*ALDOA-rs1* 10 127,345,239 - - -

*C10orf137* 10 127,408,084 *2700050L05Rik* 7 140,778,707

*MRGPRX3* 11 18,142,502 *Gm660* 7 54,564,748

- - - *Tpi1-rs13* 7 54,608,077

- - - *Tpi1-rs11* 7 54,777,639

- - - *Tpi1-rs12* 7 55,039,236

- - - *Tpi1-rs14* 7 55,246,555

*MRGPRX4* 11 18,194,384 *Mrgprx1* 7 55,276,341

- - - *Tpi1-rs10* 7 54,502,936

- - - *Tpi1-rs15* 7 54,503,234

*PTPN5* 11 18,749,475 *Ptpn5* 7 54,333,172

*PPP2R5B* 11 64,692,180 *Ppp2r5b* 19 6,227,767

*PGAM1-rs13* 11 64,698,204 - - -

*GPHA2* 11 64,701,943 *Gpha2* 19 6,226,401

*CHORDC1* 11 89,933,597 *Chordc1* 9 18,096,711

*PGAM1-rs8* 11 91,738,423 - - -

*FAT3* 11 92,085,262 *Fat3* 9 15,714,637

*PTS* 11 112,097,088 *Pts* 9 50,329,722

- - - *Pgam1-rs7* 9 50,023,246

*NCAM1* 11 112,831,995 *Ncam1* 9 49,310,243

*RICS* 11 128,834,955 *Grit* 9 32,015,785

- - - *Tpi1-rs3* 9 31,755,930

*BARX2* 11 129,245,881 *Barx2* 9 31,653,865

*SFRS2IP* 12 46,312,914 *Sfrs2ip* 15 96,242,129

*PGAM1-rs16* 12 46,570,073 - - -

*SLC38A1* 12 46,576,838 *Slc38a1* 15 96,405,106

*BTG1* 12 92,534,054 *Btg1* 10 96,079,661

*PGAM1-rs4* 12 92,557,837 - - -

*EEA1* 12 93,166,285 *Eea1* 10 95,403,297

*PLXNC1* 12 94,542,499 *Plxnc1* 10 94,255,931

*PGAM1-rs5* 12 94,590,196 - - -

*CCDC41* 12 94,702,056 *4921537D05Rik* 10 94,151,535

*IGF1* 12 102,789,645 *Igf1* 10 87,322,146

*PGAM1-rs1* 12 102,948,637 - - -

*PAH* 12 103,232,104 *Pah* 10 86,984,714

*PCDH17* 13 58,205,789 *Pcdh17* 14 84,845,481

- - - *Pgk1-rs11* 14 86,629,502

- - - *Pgk1-rs10* 14 86,629,605

*PGAM1-rs15* 13 59,794,725 - - -

*DIAPH3* 13 60,239,717 *Diap3* 14 87,056,130

*TSHR* 14 81,421,869 *Tshr* 12 92,639,491

- - - *Pgam5-rs1* 12 92,720,490

*GTF2A1* 14 81,646,394 *Gtf2a1* 12 92,797,155

*SEL1L* 14 81,939,239 *Sel1l* 12 93,044,483

- - - *Pgk1-rs7* 12 94,954,868

*FLRT2* 14 85,996,488 *Flrt2* 12 96,930,464

*SEMA4B* 15 90,728,152 *Sema4b* 7 87,331,727

*ENO1-rs2* 15 90,765,047 - - -

*ENO1-rs3* 15 90,766,554 - - -

*CIB1* 15 90,773,477 *Cib1* 7 87,372,046

*RGMA* 15 93,586,637 *Rgma* 7 80,520,393

*PGAM1-rs2* 15 94,784,888 - - -

*MCTP2* 15 94,841,495 *Mctp2* 7 79,222,716

*RAB11FAP4* 17 29,718,642 *Rab11fap4* 11 79,404,714

- - - *Pgam1-rs4* 11 79,710,680

*UTP6* 17 30,190,190 *Utp6* 11 79,747,458

*DSG3* 18 29,027,732 *Dsg3* 18 20,668,805

- - - *Tpi1-rs4* 18 20,706,529

*DSG2* 18 29,078,027 *Dsg2* 18 20,716,608

*BRUNOL4* 18 34,823,003 *Brunol4* 18 25,637,616

- - - *Hk1-rs1* 18 29,504,342

*PIK3C3* 18 39,535,199 *Pik3c3* 18 30,432,550

*C18orf22* 18 77,794,358 *1110032A13Rik* 18 80,389,003

- - - *Pgam1-rs5* 18 80,359,524

*ADNP2* 18 77,866,915 *Zfp508* 18 80,322,474

*SERTAD3* 19 40,946,748 *Sertad3* 7 28,258,859

- - - *Pgam1-rs1* 7 28,253,905

*BLBRB* 19 40,953,691 *Blbrb* 7 28,232,997

*PRKCSH* 20 11,546,269 *Prkcsh* 9 21,807,479

*PGAM1-rs11* 20 11,549,802 - - -

*ELAVL3* 20 11,562,143 *Elavl3* 9 21,822,220

*NCAM2* 21 22,370,633 *Ncam2* 16 81,201,016

- - - *Pgk1-rs8* 16 81,909,252

*MRPL39* 21 26,957,968 *Mrpl39* 16 84,717,823

*CXorf36* X 45,007,618 *4930578C19Rik* X 17,995,553

*PGAM1-rs9* X 46,390,044 - - -

*CHST7* X 46,433,192 *Chst7* X 19,636,696

*GNL3L* X 54,556,644 *Gnl3l* X 147,417,684

*PGAM1-rs12* X 54,715,275 - - -

*MAGED2* X 54,834,171 *Maged2* X 147,240,964

*HEPH* X 65,382,663 *Heph* X 93,650,800

*PKM2-rs3* X 65,634,319 - - -

*EDA2R* X 65,815,479 *Eda2r* X 94,531,258

*AR* X 66,763,874 *Ar* X 95,345,089

*PGK1-rs1* X 67,206,648 - - -

*OPHN1* X 67,262,186 *Ophn1* X 95,752,854

*MAGT1* X 77,081,861 *2610529C04Rik* X 103,165,556

*PGAM1-rs7* X 77,110,148 - - -

*COX7B* X 77,154,961 *Cox7b* X 103,211,039

*TBX22* X 79,270,255 *Tbx22* X 104,863,303

*HK2-rs1* X 79,711,597 - - -

*BRWD3* X 79,931,683 *Brwd3* X 105,937,547

*RBM41* X 106,310,368 *Rbm41* X 136,478,780

- - - *Pgam1-rs9* X 136,493,319

*CXorf41* X 106,449,862 *E230019M04Rik* X 136,597,418
