# Supplementary material for: Frequent and recent retrotransposition of orthologous genes plays a role in the evolution of sperm glycolytic enzymes
Source: BMC Genomics. 2010 May 6;11:285. doi: 10.1186/1471-2164-11-285 (PMC2881024; doi:10.1186/1471-2164-11-285)
Supplement: Additional file 4 — Amino acid alignment of retroposed sequences in the human genome that maintain ORFs. Asterisks (*) denote identical residues. Methionine residues are highlighted in grey boxes, residues marked as "X" in a black box denote stop codons, and dashes denote deleted codons. [file 1471-2164-11-285-S4.PDF]

```
TPI1        MAEDGEEAEFHFAALYISGQWPRLRADTDLQRLGSSAMAPSRKFFVGGNWKMNGRKQSLGELIGTLNAAKVPADTEVVCAPPTAYIDFARQKLDPKIAVA  100
TPI1-rs1    --------------------XESYCR*********************************R**VR***************T********************   79

TPI1        AQNCYKVTNGAFTGEISPGMIKDCGATWVVLGHSERRHVFGESDELIGQKVAHALAEGLGVIACIGEKLDEREAGITEKVVFEQTKVIADNVKDWSKVVL  200
TPI1-rs1    ************************R***************************************T***********************************  179

TPI1        AYEPVWAIGTGKTATPQQAQEVHEKLRGWLKSNVSDAVAQSTRIIYGGSVTGATCKELASQPDVDGFLVGGASLKPEFVDIINAKQX               286
TPI1-rs1    ******************D****D**************************************G***********************                265


PGAM1       MAAYKLVLIRHGESAWNLENRFSGWYDADLSPAGHEEAKRGGQALRDAGYEFDICFTSVQKRAIRTLWTVLDAIDQMWLPVVRTWRLNERHYGGLTGLNK  100
PGAM1-rs7   **************T********C*******************************L******V*************************************  100

PGAM1       AETAAKHGEAQVKIWRRSYDVPPPPMEPDHPFYSNISKDRRYADLTEDQLPSCESLKDTIARALPFWNEEIVPQIKEGKRVLIAAHGNSLRGIVKHLEGL  200
PGAM1-rs7   ****************************************************Y**P**********************************Q**A**V***  200

PGAM1       SEEAIMELNLPTGIPIVYELDKNLKPIKPMQFLGDEETVRKAMEAVAAQGKAKKX                                               254
PGAM1-rs7   ***************************************C**I***********                                                254


ENO1        MSILKIHAREIFDSRGNPTVEVDLFTSKGLFRAAVPSGASTGIYEALELRDNDKTRYMGKGVSKAVEHINKTIAPALVSKKLNVTEQEKIDKLMIEMDGT  100
ENO1-rs1    **********L****************E*****************V***Q**************P**P*******V************************  100

ENO1        ENKSKFGANAILGVSLAVCKAGAVEKGVPLYRHIADLAGNSEVILPVPAFNVINGGSHAGNKLAMQEFMILPVGAANFREAMRIGAEVYHNLKNVIKEKY  200
ENO1-rs1    *****************A***S*********H*****S***K******V******S***VT********V************P*******S*********  200

ENO1        GKDATNVGDEGGFAPNILENKEGLELLKTAIGKAGYTDKVVIGMDVAASEFFRSGKYDLDFKSPDDPSRYISPDQLADLYKSFIKDYPVVSIEDPFDQDD  300
ENO1-rs1    *****G***G*A*****************************IVS***E************E**FL***T******C**********N*****T*******  300

ENO1        WGAWQKFTASAGIQVVGDDLTVTNPKRIAKAVNEKSCNCLLLKVNQIGSVTESLQACKLAQANGWGVMVSHRSGETEDTFIADLVVGLCTGQIKTGAPCR  400
ENO1-rs1    ****************E***R******T*S*****K***********R*****************C***P*H*****N***T******XP**L******X  398

ENO1        SERLAKYNQLLRIEEELGSKAKFAGRNFRNPLAKX                                                                   434
ENO1-rs1    ******************************P***                                                                    432
```
