# Supplementary material for: Frequent and recent retrotransposition of orthologous genes plays a role in the evolution of sperm glycolytic enzymes
Source: BMC Genomics. 2010 May 6;11:285. doi: 10.1186/1471-2164-11-285 (PMC2881024; doi:10.1186/1471-2164-11-285)
Supplement: Additional file 5 — Amino acid alignment of GPI1-related sequences in the mouse genome that maintain ORFs (GPI1-rs1). Asterisks (*) denote identical residues. Methionine residues are highlighted in grey boxes, dashes denote deleted codons and the stop codon is marked as "X" in a black box. [file 1471-2164-11-285-S5.PDF]

```
Gpi1       LRVPLGSLAMAALTRNPQFQKLLEWHRANSANLKLRELFEADPERFNNFSLNLNTNHGHILVDYSKNLVNKEVMQMLVELAKSRGVEAARDNMFSGSKINY 100
Gpi1-rs1   MSCSVYLSGPSPWL*S*GTRSSRSCWSG--T*********************************************************************  98

Gpi1       TENRAVLHVALRNRSNTPIKVDGKDVMPEVNRVLDKMKSFCQRVRSGDWKGYTGKSITDIINIGIGGSDLGPLMVTEALKPYSKGGPRVWFVSNIDGTHIA 200
Gpi1-rs1   **D*******************V*******************---------------------------------------------------------- 140

Gpi1       KTLASLSPETSLFIIASKTFTTQETITNAETAKEWFLEAAKDPSAVAKHFVALSTNTAKVKEFGIDPQNMFEFWDWVGGRYSLWSAIGLSIALHVGFDHFE 300
Gpi1-rs1   -----------------*L******************************************************************T*P************ 223

Gpi1       QLLSGAHWMDQHFLKTPLEKNAPVLLALLGIWYINCYGCETHALLPYDQYMHRFAAYFQQGDMESNGKYITKSGARVDHQTGPIVWGEPGTNGQHAFYQLI 400
Gpi1-rs1   **************************************************Y************************************************* 323

Gpi1       HQGTKMIPCDFLIPVQTQHPIRKGLHHKILLANFLAQTEALMKGKLPEEARKELQAAGKSPEDLEKLLPHKVFEGNRPTNSIVFTKLTPFILGALIAMYEH 500
Gpi1-rs1   ******************************************M********************************************************* 423

Gpi1       KIFVQGIMWDINSFDQWGVELGKQLAKKIEPELEGSSAVTSHDSSTNGLISFIKQQRDTKLEX                                      562
Gpi1-rs1   ********************************************************W******                                      485
```
