# Supplementary material for: Frequent and recent retrotransposition of orthologous genes plays a role in the evolution of sperm glycolytic enzymes
Source: BMC Genomics. 2010 May 6;11:285. doi: 10.1186/1471-2164-11-285 (PMC2881024; doi:10.1186/1471-2164-11-285)
Supplement: Additional file 6 — Amino acid alignments are shown for retroposed sequences containing upstream start codons. (A) Amino acid sequence alignment comparing upstream extensions of human retroposed sequences to their parent glycolytic enzymes. (B) Amino acid sequence alignment of mouse retroposed sequences with upstream start codons and their parent glycolytic enzymes. Asterisks (*) denote identical residues. Methionine residues are highlighted in grey boxes, residues marked as "X" in a black box denote stop codons, and dashes indicate deleted codons. Pkm2-Ss sequence is from [8]. Hs (Homo sapiens), Bt (Bos taurus), Ss (Su scrofa), Mm (Mus musculus). [file 1471-2164-11-285-S6.PDF]

A.

```
PGK1-Hs         LPSCISKSARRQSAPSLTESPTSLPSCISKMSLSN 35
PGK1-rs1        MEIR*LQASQAHISSR*PYCLNHR*LS******** 35

PGAM1-Hs        VPHPQPAAMAAYK 13
PGAM1-rs6       MLS*K**T**T** 13
```

B.

```
Tpi1-Mm         KAEQQGAGLTMAEGGEKEEFCFTAIYISGQWREPCVCTDLQRLEPGTMAPTRK 53
Tpi1-rs5        M*HIHRHISHTHTHTHTHTHTHTHTL*LWKGHSLYCDS******LSA***S** 53

Eno1_Mm         RLSSVSTAPSFLALQRSYCQKFAMSILR 28
Eno1-rs5        M*****IE****P****D****T*T*VN 28
Eno1-rs9            M**E****PA***S**M*T*ATSF 24

PKM2-Hs         EGGAAEGLRRPAAGXGGSGSLHSSCTRRGSGSLRLCSVARVGQRRRTSAAMSKPH 54
Pkm2-Bt         GDPQSGXPXGXGS*SPHWSDASRAPGPVC*SRASRAGPRRSSSGPK**E****H* 53
Pkm2-Ss                                                       **E**P*A*  9
Pkm2-Mm         AAEGLRRPAAVITLRPSRR*PAQQRLVFT*LTSA*GIA*GTEV*P***GT*P*** 54
Pkm2-rs1                      MPTRR*PAQQRPVLARQTSA*GIT*GTEV*L***GT*P*** 41
Pkm2-rs2                                       MSA*GIT*GTKVHP***GTTP*** 22
Pkm2-rs3                                       MSA*GIT*GTKVHP***GTTP*** 22
Pkm2-rs4                                            MA*ECRVCL***ET*P*** 15
Pkm2-rs8        MIEPFKEDINNPLKEKTGKRP-QQRCILTWQT*T*GIA*GTKVYP***GT*Q*** 54
```
