# Supplementary figures and images for: Frequent and recent retrotransposition of orthologous genes plays a role in the evolution of sperm glycolytic enzymes
Source: BMC Genomics. 2010 May 6;11:285. doi: 10.1186/1471-2164-11-285 (PMC2881024; doi:10.1186/1471-2164-11-285)

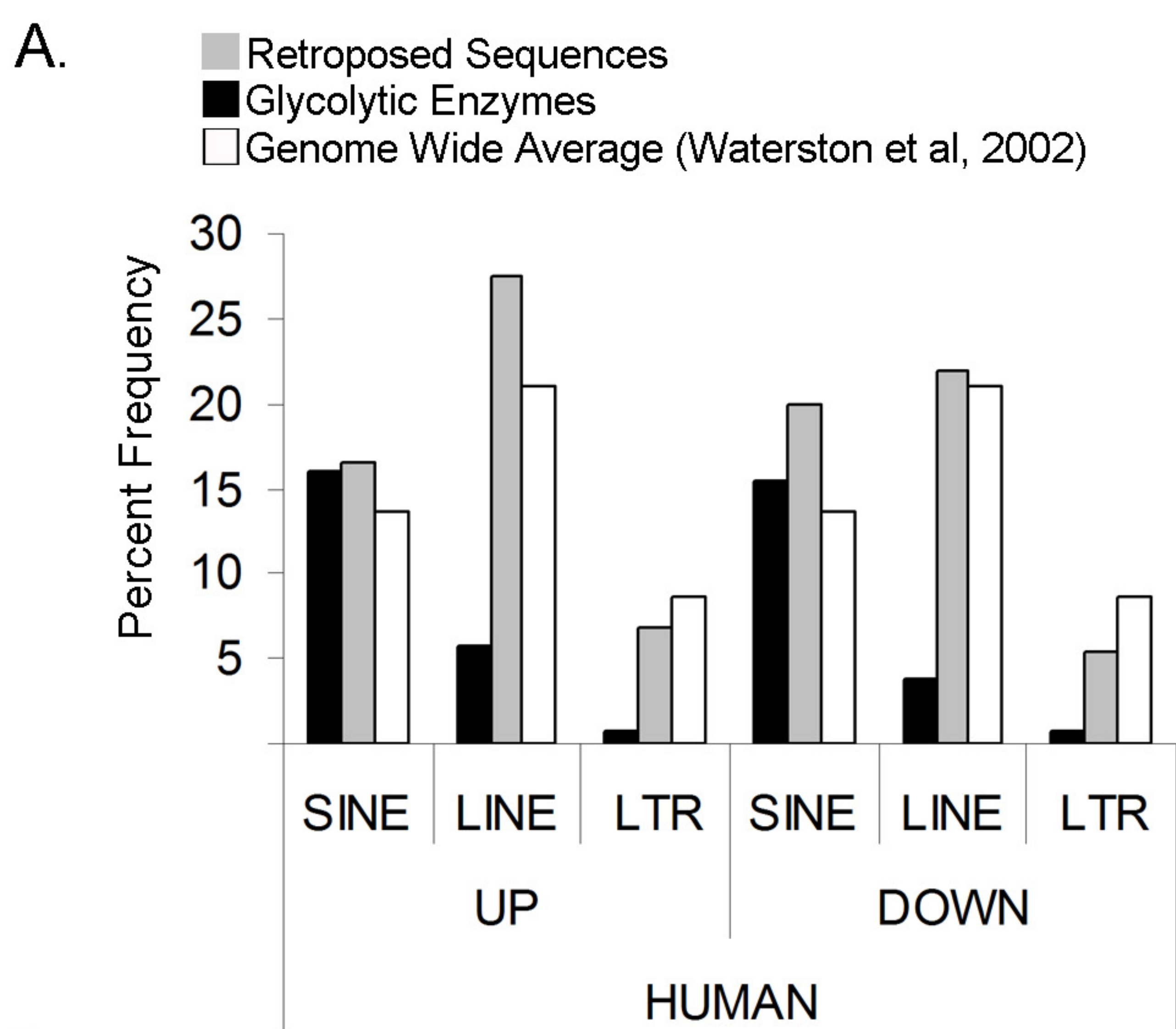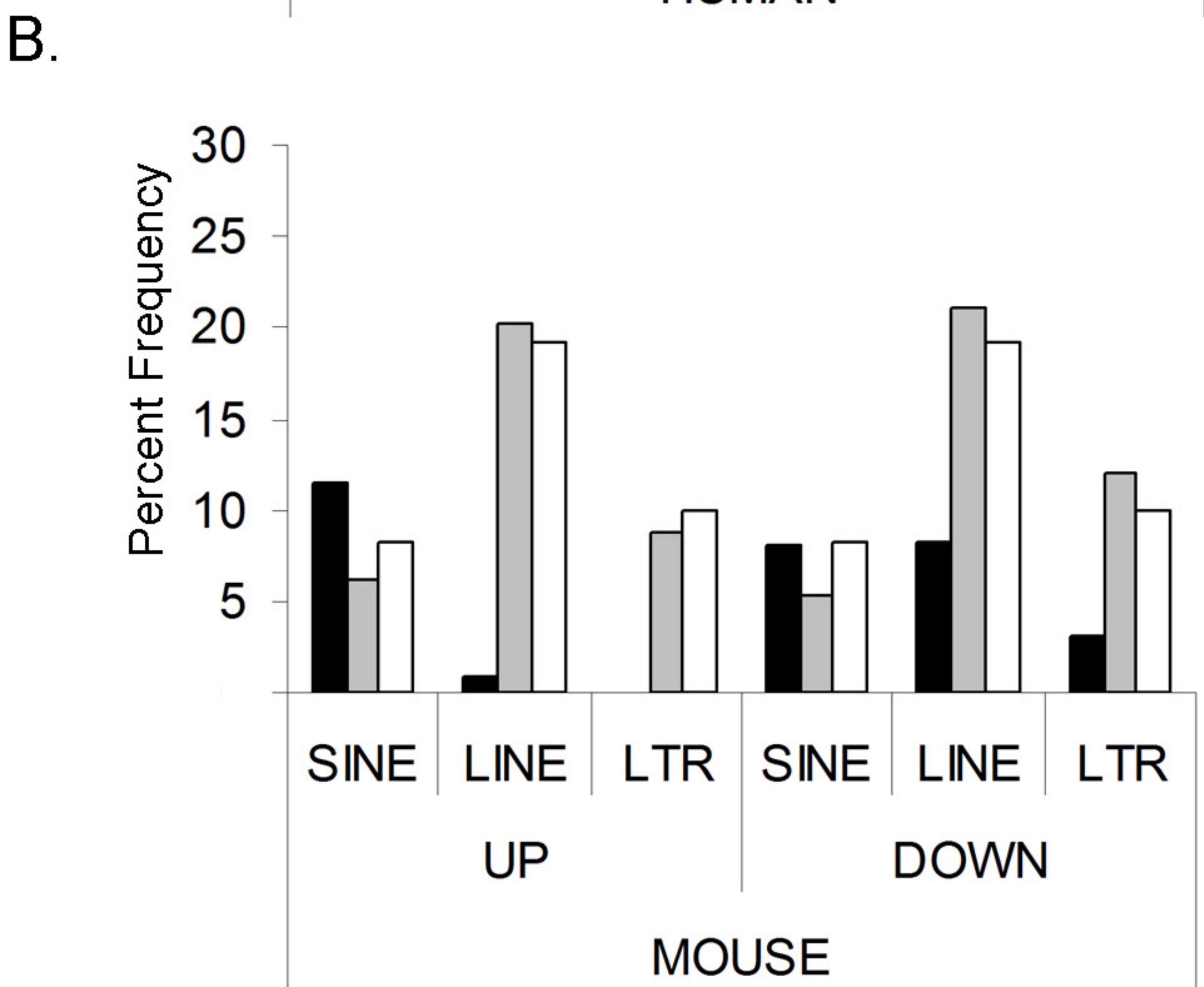

Supplement: Additional file 7 — Percent frequency of repetitive elements flanking retroposed sequences and genes encoding glycolytic enzymes in the (A) human and (B) mouse genomes. Black bars represent the percent frequency of SINE, LINE and LTR elements flanking all intron-containing parent genes that encode glycolytic enzymes. Gray bars denote the percent frequency of SINE, LINE and LTR elements both upstream and downstream of retroposed sequences derived from these parent genes. White bars represent the genome average frequency of these elements, as determined by Waterston et al., 2002 [45]. [file 1471-2164-11-285-S7.PDF]
