# Supplementary material for: Frequent and recent retrotransposition of orthologous genes plays a role in the evolution of sperm glycolytic enzymes
Source: BMC Genomics. 2010 May 6;11:285. doi: 10.1186/1471-2164-11-285 (PMC2881024; doi:10.1186/1471-2164-11-285)
Supplement: Additional file 8 — Retroposed and non-retroposed genes evolve at similar rates within each gene family. The figure shows the phylogenetic trees for five gene families (glyceraldehyde 3-phosphate dehydrogenase, pyruvate kinase, aldolase, phosphoglycerate mutase and hexokinase). h, denotes human genes; m, denote mouses genes. Black lines are used in branches for which we did not find evidence of retrotransposition. Red lines represent branches with evidence of retrotransposition. Numbers denote the average branch length since the primate/rodent split. [file 1471-2164-11-285-S8.PDF]

Glyceraldehyde 3-phosphate dehydrogenase

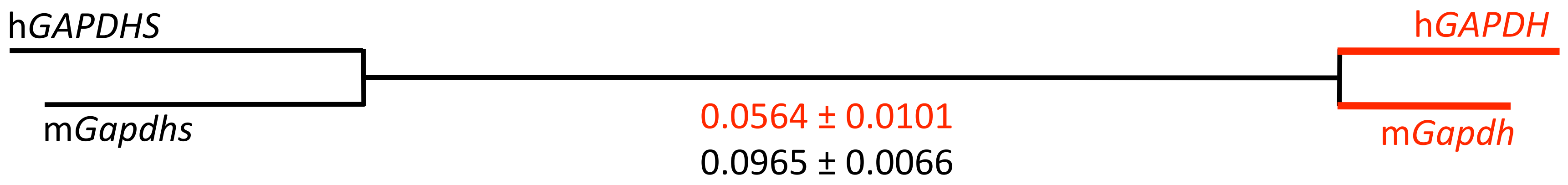

Pyruvate kinase

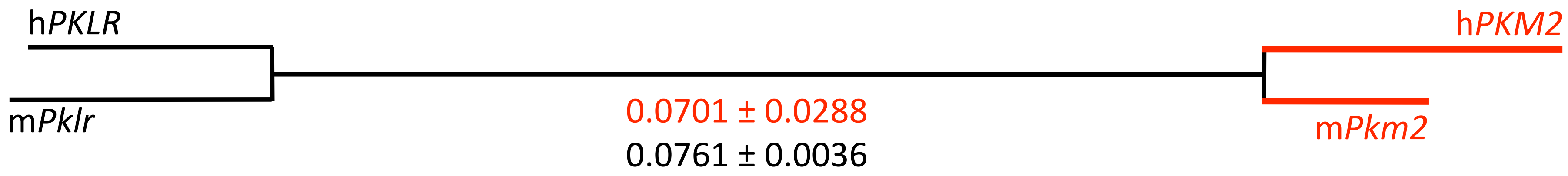

Aldolase

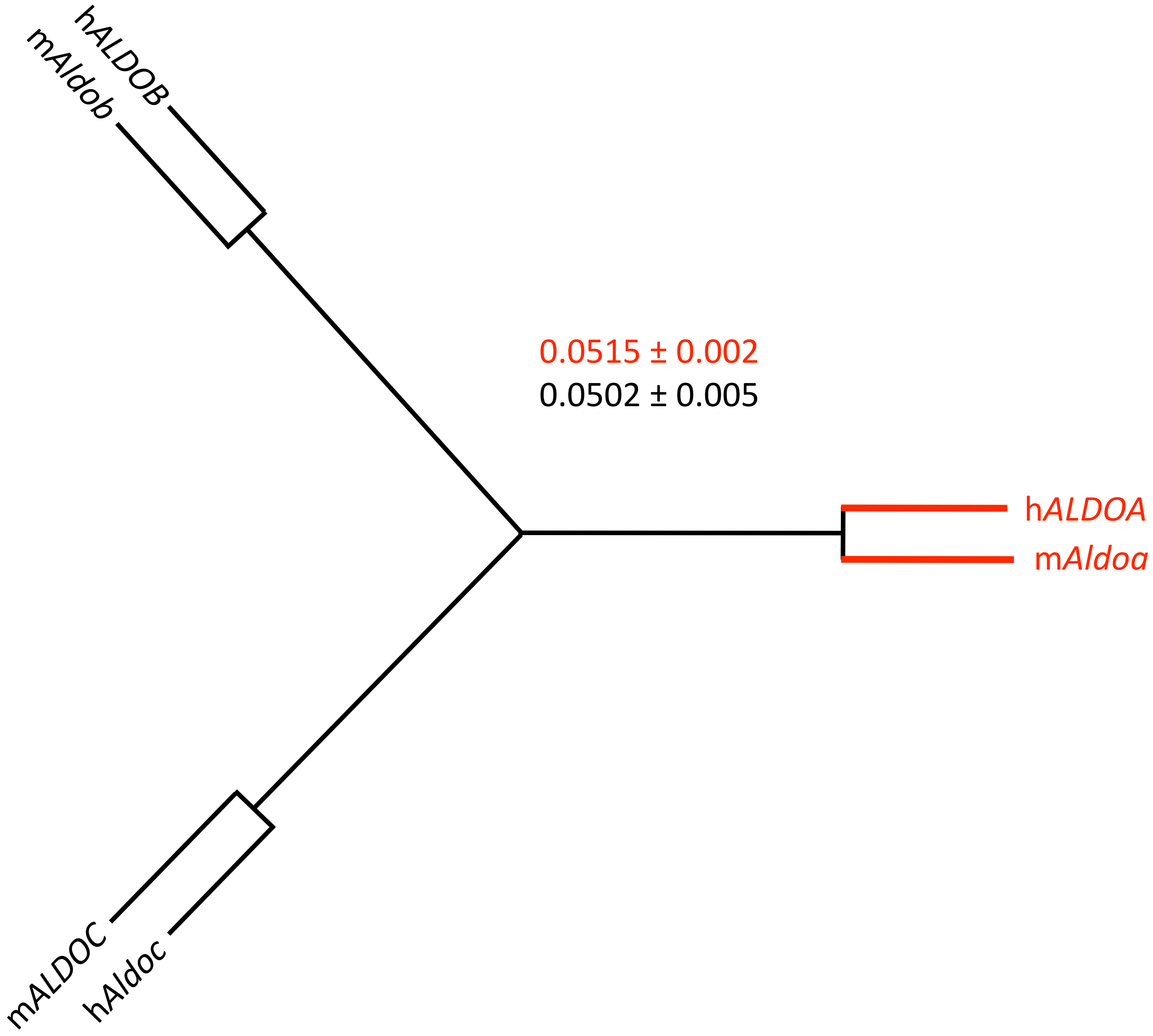

hALDOB
mAldob
0.0515 ± 0.002
0.0502 ± 0.005
hALDOA
mAldoa
mALDOC
hAldoc

Phosphoglycerate mutase

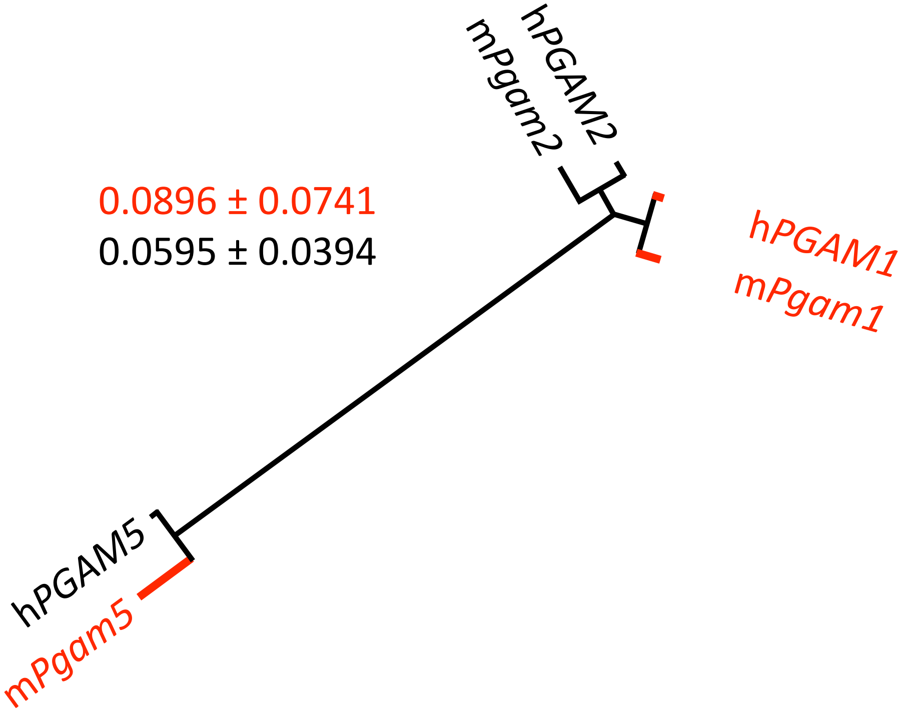

Hexokinase

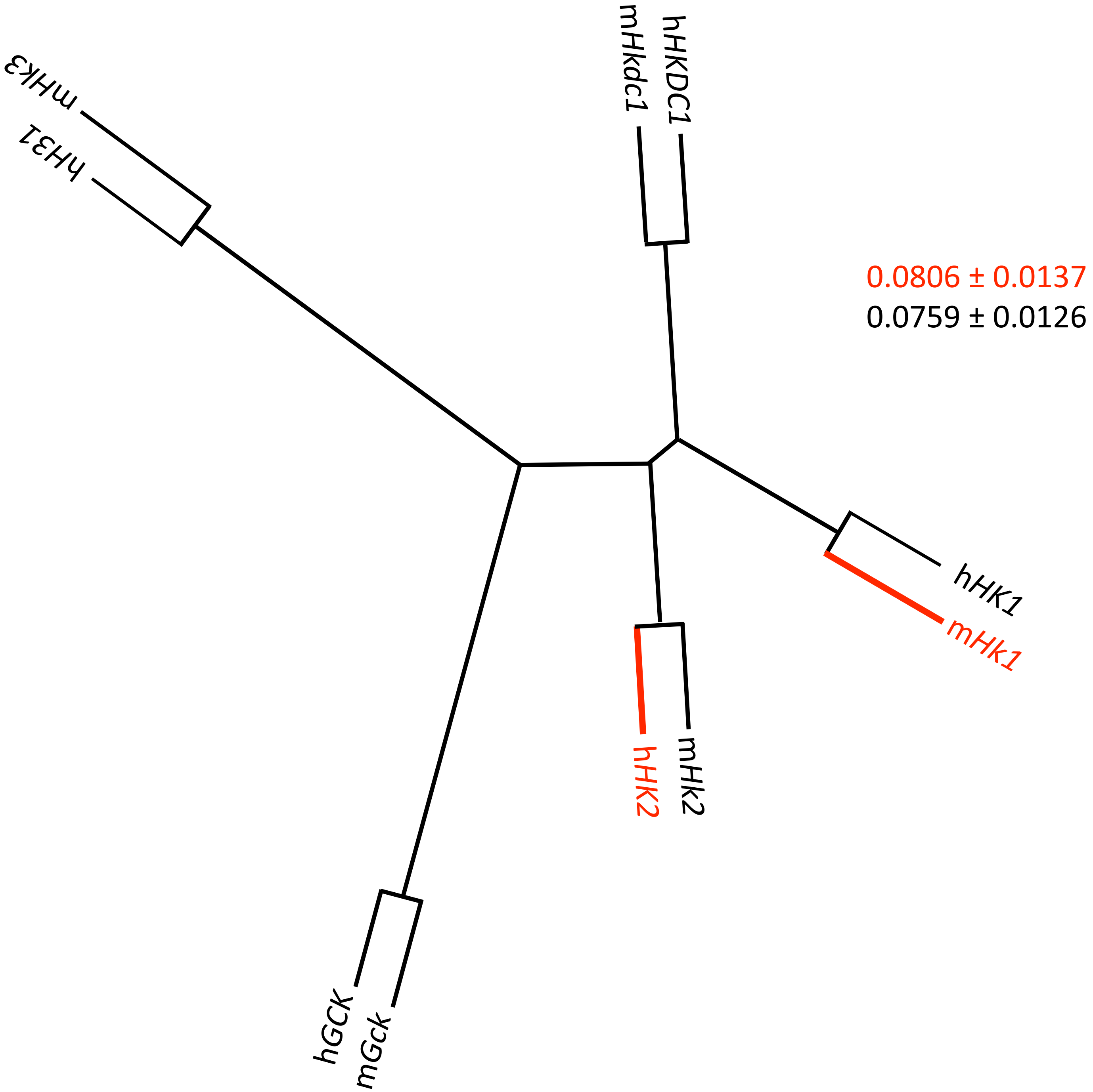
mHk3
hHK3
mHkdc1
hHKDC1
0.0806 ± 0.0137
0.0759 ± 0.0126
hHK1
mHk1
hHK2
mHk2
hGCK
mGck
